# Integrated proteogenomic and metabolomic profiling of acute myeloid leukemias to identify molecular subtypes and associated therapy targets

**DOI:** 10.1038/s43018-026-01175-6

**Published:** 2026-06-12

**Authors:** Shih-Chun A. Chu, Yi Hsiao, Chenwei Wang, Jennifer E. Kyle, Raghav Jain, Yamei Deng, Marina A. Gritsenko, Leanne E. Henry, Jonathan T. Lei, Yongchao Dou, Bahar Tercan, Zhiao Shi, Mahnoor N. Gondal, Chia-Feng Tsai, John M. Elizarraras, Rosalie K. Chu, Fengchao Yu, Sunil K. Joshi, Xiaojun Jing, Daniel A. Polasky, Karl K. Weitz, Ginny Xiaohe Li, Vanessa L. Paurus, Chaevien S. Clendinen, Athena A. Schepmoes, Priscila M. Lalli, Josie G. Eder, Javier E. Flores, Kelly G. Stratton, James C. Pino, Camilo Posso, Vladislav A. Petyuk, Tyler J. Sagendorf, Yuanwei Xu, Omar M. Ibrahim, Ronald J. Moore, Rui Zhao, Jin Chen, Matthew E. Monroe, Mathangi Thiagarajan, Galen Hostetter, Chelsea Newton, Eunkyung An, Ana I. Robles, Xu Zhang, Nathan J. Edwards, Yin Lu, Hui Zhang, Haitham Abdelhakim, Paul D. Piehowski, Mehdi Mesri, Richard D. Smith, Chandan Kumar-Sinha, Cristina E. Tognon, Jennifer Dunlap, Elie Traer, Li Ding, Jeffrey W. Tyner, Arul M. Chinnaiyan, Gilbert S. Omenn, Karin D. Rodland, Saravana M. Dhanasekaran, Sara J. C. Gosline, Alexey I. Nesvizhiskii, Bing Zhang, Tao Liu, Marcin P. Cieslik, Clinical Proteomic Tumor Analysis Consortium, Shih-Chun A. Chu, Jennifer E. Kyle, Raghav Jain, Yamei Deng, Marina A. Gritsenko, Leanne E. Henry, Jonathan T. Lei, Yongchao Dou, Bahar Tercan, Zhiao Shi, Mahnoor N. Gondal, John M. Elizarraras, Fengchao Yu, Sunil K. Joshi, Xiaojun Jing, Daniel A. Polasky, Karl K. Weitz, Ginny Xiaohe Li, Vanessa L. Paurus, Athena A. Schepmoes, Priscila M. Lalli, Josie G. Eder, Javier E. Flores, Kelly G. Stratton, James C. Pino, Camilo Posso, Vladislav A. Petyuk, Tyler J. Sagendorf, Omar M. Ibrahim, Ronald J. Moore, Rui Zhao, Jin Chen, Matthew E. Monroe, Galen Hostetter, Chelsea Newton, Eunkyung An, Ana I. Robles, Xu Zhang, Nathan J. Edwards, Yin Lu, Hui Zhang, Haitham Abdelhakim, Paul D. Piehowski, Mehdi Mesri, Richard D. Smith, Chandan Kumar-Sinha, Cristina E. Tognon, Jennifer Dunlap, Elie Traer, Li Ding, Jeffrey W. Tyner, Arul M. Chinnaiyan, Gilbert S. Omenn, Karin D. Rodland, Saravana M. Dhanasekaran, Sara J. C. Gosline, Alexey I. Nesvizhiskii, Bing Zhang, Tao Liu, Marcin P. Cieslik, Shelby Abts, Anupriya Agarwal, Veera Baladandayuthapani, Anand Basu, Garana Belinda, William Bocik, Melissa Borucki, Shuang Cai, Stancioaica Maria Camelia, Steven Carr, Patricia Castro, Daniel Chan, Hanbyul Cho, Rosalie K. Chu, Chaevien S. Clendinen, Simona Colantonio, Reese Crispen, Diwaker Davar, Rajiv Dhir, Marcin Domagalski, John Evangelista, Brenda Fevrier-Sullivan, Rafael Fonseca, John Freymann, Victoria Fulidou, Sharon Gaheen, Pencho Georgiev, Gad Getz, Lidia Gil, Michael A. Gillette, Andrew K. Godwin, Charles A. Goldthwaite, Vladislav Golubkov, Ramaswamy Govindan, Anthony Green, Michael Holck, Noshad Hosseini, Yi Hsiao, Joel Hsu, Lan Huang, Michael Ittmann, Eric Jaehnig, Karen A. Ketchum, Justin Kirby, Iga Kolodziejczak, Yelena V. Krutikova, Toan Le, Qing Kay Li, T. Mamie Lih, Avi Ma’ayan, Micheal J. MacCoss, Kiran K. Mangalaparthi, D. R. Mani, Rahul Mannan, Monica Mays, Peter McGarvey, James Noyama, Kristen Nyce, Akhilesh Pandey, Abhijit Parolia, Amanda G. Paulovich, Francesca Petralia, Alex Pico, Alexander Pilozzi, Pinar O. Eser, Olga Potapova, Marina Prilutskaya, Gustavo Rivero, Dan Rohrer, Paul Rudnick, Shankha Satpathy, Yvonne Shutack, Sandra S. Garcia-Buntley, Ratna R. Thangudu, Mathangi Thiagarajan, Chia-Feng Tsai, Negin Vatanian, Sudha Venkatachari, Miodrag Vucic, Chenwei Wang, Pei Wang, Yuefan Wang, Bart O. Williams, Maciej Wiznerowicz, Yuanwei Xu, Kakhaber Zaalishvili

**Affiliations:** 1 Department of Pathology, University of Michigan, Ann Arbor, MI USA; 2 Gilbert S. Omenn Department of Computational Medicine and Bioinformatics, University of Michigan, Ann Arbor, MI USA; 3 Michigan Center for Translational Pathology, University of Michigan, Ann Arbor, MI USA; 4 Lester and Sue Smith Breast Center, Baylor College of Medicine, Houston, TX USA; 5 Department of Molecular and Human Genetics, Baylor College of Medicine, Houston, TX USA; 6 Biological Sciences Division, Pacific Northwest National Laboratory, Richland, WA USA; 7 Environmental and Molecular Sciences Division, Pacific Northwest National Laboratory, Richland, WA USA; 8 Institute for Systems Biology, Seattle, WA USA; 9 Texas Children’s Hospital, Department of Pediatrics, Houston, TX USA; 10 Dan L. Duncan Cancer Center, Baylor College of Medicine, Houston, TX USA; 11 Department of Medicine, Divisions of Hematology and Oncology, Stanford University School of Medicine, Stanford, CA USA; 12 Cancer Institute, Stanford University School of Medicine, Stanford, CA USA; 13 Department of Pathology. Johns Hopkins University School of Medicine, Baltimore, MD USA; 14 Department of Medicine, Washington University in St. Louis, St. Louis, MO USA; 15 McDonnell Genome Institute, Washington University in St. Louis, St. Louis, MO USA; 16 Office of Cancer Clinical Proteomics Research, Division of Cancer Treatment and Diagnosis, National Cancer Institute, Rockville, MD USA; 17 Van Andel Research Institute, Grand Rapids, MI USA; 18 Department of Biochemistry and Molecular and Cellular Biology, Georgetown University Medical Center, Washington DC, USA; 19 ICF, Rockville, MD USA; 20 University of Kansas Medical Center, The University of Kansas Cancer Center, Kansas City, KS USA; 21 Knight Cancer Institute, Oregon Health and Science University, Portland, OR USA; 22 Department of Pathology, Oregon Health and Science University, Portland, OR USA; 23 Division of Hematology and Medical Oncology, Department of Medicine, Oregon Health and Science University, Portland, OR USA; 24 Division of Oncology, Department of Medicine, Washington University, St. Louis, MO USA; 25 Siteman Cancer Center, Washington University, St. Louis, MO USA; 26 Department of Cell, Developmental and Cancer Biology, Knight Cancer Institute, Oregon Health and Science University, Portland, OR USA; 27 Department of Urology, University of Michigan Medical School, Ann Arbor, MI USA; 28 Howard Hughes Medical Institute, Ann Arbor, MI USA; 29 Department of Cell, Developmental and Cancer Biology, Oregon Health and Science University, Portland, OR USA; 30 Department of Biomedical Engineering, Oregon Health and Sciences University, Portland, OR USA; 31 School of Public Health, University of Michigan, Ann Arbor, MI USA; 32 Frederick National Laboratory for Cancer Research, Frederick, MD USA; 33 Leidos Biomedical Research, Inc., Huntsville, AL USA; 34 Fidelis Research, Sofia, Bulgaria; 35 Broad Institute of MIT and Harvard, Cambridge, MA USA; 36 University of Pittsburgh Medical Center, Pittsburgh, PA USA; 37 Icahn School of Medicine at Mount Sinai, New York, NY USA; 38 Department of Laboratory Medicine and Pathology, Mayo Clinic, Rochester, MN USA; 39 International Institute for Molecular Oncology, Poznań, Poland; 40 Cureline Human Biospecimen CRO, South San Francisco, CA USA; 41 University of California, Irvine, CA USA; 42 ProteoGenex, Inc., Culver City, CA USA; 43 University of Washington, Seattle, WA USA; 44 Fred Hutch Cancer Center, Seattle, WA USA; 45 Gladstone Institutes, San Francisco, CA USA; 46 TGH Cancer Center, Tampa, FL USA; 47 Spectragen Informatics, Bainbridge Island, WA USA; 48 BioPartners, Westlake Village, CA USA

**Keywords:** Cancer genomics, Mass spectrometry, Proteomic analysis, Predictive markers, Cancer

## Abstract

Acute myeloid leukemia (AML) is a genetically and phenotypically heterogeneous hematological malignancy. Here, to better define this clinically taxing and translationally challenging malignancy, we applied a multiomics approach, consisting of 13 modalities to analyze 173 treatment-naive individuals with AML. By integrating these ‘omes’, we identified distinct AML subtypes, genotype–phenotype associations, biomarkers and pathobiological mechanisms. Across the spectrum of primitive and committed AML, we found extensive metabolomic and lipidomic reprogramming driven by divergent MYC and mTOR activity. We linked metabolic changes to striking hyperacetylation of mitochondrial proteins in *CEBPA*-mutant AML. Protein-centric subtyping revealed a distinct *NPM1*-mutant subset characterized by outlier expression of FOXC1 and HOXB8/9. To nominate therapeutic targets across subtypes, we developed a multiomic machine-learning approach and validated MTA1 as a contributor to panobinostat resistance. Altogether our findings underscore the complex nature of AML and provide a clinically and translationally informed unified view that reveals coalescent phenotypes across multiomic layers.

## Abstract (web summary)

Chu et al. conducted multiomic analyses of samples from 173 treatment-naive individuals with acute myeloid leukemia, integrating proteogenomic and metabolomic modalities to identify subtypes, and developed a machine-learning approach to find therapy targets.

## Main

Acute myeloid leukemia (AML) is a complex and heterogeneous hematologic malignancy characterized by rapid proliferation of myeloblasts in the bone marrow. Despite recent advancements in therapeutic options, it is still one of the most clinically challenging blood cancers, with a notably low 5-year survival rate of 30% and a high rate of relapse^1,2^. While intensive chemotherapy and allogeneic hematopoietic stem cell (HSC) transplantation remain the front-line treatment option for most persons, recent US Food and Drug Administration (FDA)-approved targeted therapeutics are increasingly used to treat persons with specific genetic aberrations, such as *IDH1* or *FLT3* internal tandem duplication (ITD) mutations. Phenotypic characterization of tumors has shown promise in preclinical studies^3–5^, setting the stage for further advances in guiding therapies through integrative genetic and phenotypic stratification^6^.

AML can develop de novo or from an antecedent diagnosis of myelodysplastic syndrome (MDS) or myeloproliferative neoplasm. This varied ontogeny is reflected in distinct mutational profiles^7^, histological features and molecular phenotypes, resulting in complex classification schemes aimed at different clinical objectives^8^. Important classifications based on cytogenetics and mutations are from the European Leukemia Net (ELN)^2^ and the World Health Organization (WHO)^9^. These are supplemented by morphological classifications such as the French–American–British (FAB)^10^ system and emerging efforts at molecular subtyping that delineate, among others, primitive, committed and granulocyte-macrophage progenitor (GMP)-like AML subtypes^11^. However, no single classification captures the extent of AML clinical heterogeneity^12^, underscoring the need for tumor classification beyond established genetic features.

Recent efforts to characterize the proteogenomic landscapes of AML^13–19^ represent critical advances toward bridging AML genotypes and clinical outcomes. These studies have identified, among others, proteomic AML subsets with high mitochondrial content and sensitivity to venetoclax^13^. However, despite a growing interest in mapping the post-translational^20^, metabolic^21^ and lipidomic^22,23^ landscapes of AML, a unified multiomic classification that can be clinically exploited is currently lacking. Moreover, the extent to which recurrent genetic aberrations and common phenotypic differentiation hierarchies influence the post-translational and metabolic state of AML remains largely unknown.

To connect AML genetics with pathobiology, we generated a multiomic atlas of 173 treatment-naive individuals, spanning genomics, methylomics, transcriptomics, proteomics, post-translational modifications (PTMs), metabolomics and lipidomics. We identified eight distinct subtypes (AML-8) linking genetic drivers to specific molecular traits and drug responses. This resource exposes biological features missed by traditional profiling, offering the community a robust framework for advanced stratification and therapeutic development.

## Results

### A comprehensive proteogenomic and metabolomic compendium of AML

#### Sequencing and mass spectrometry (MS)-based multiomic characterization of treatment-naive AML

**Fig. 1:**
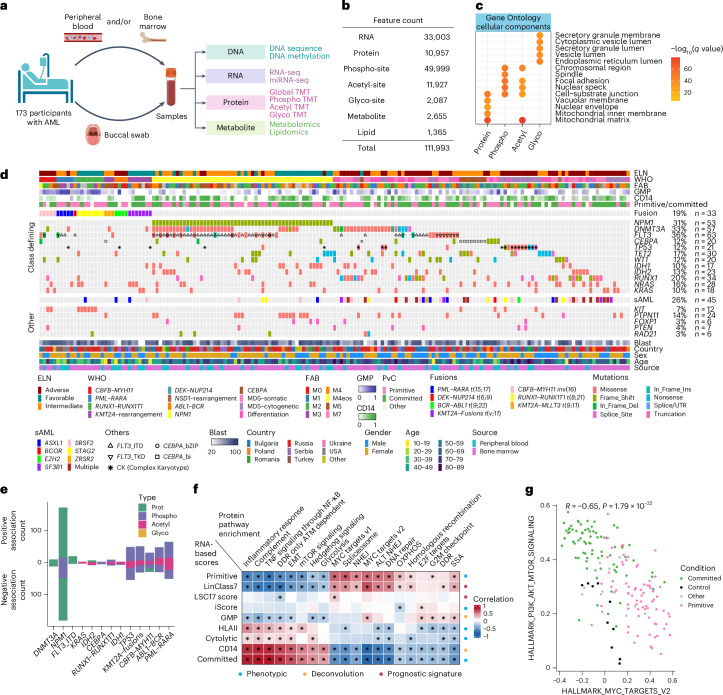
Comprehensive multiomic profiling of AML reveals divergent activity of MYC and mTOR. **a**, Schematic of sample collection and multiomic profiling of primary AML samples. **b**, Number of quantitative molecular features identified across data types. **c**, Enrichment of proteins in different assays for gene sets from the GO cellular components. **d**, Clinicogenomic landscape. Top, annotations of clinical classifications and phenotypic scores. Middle, oncoprint of class-defining and selected genetic aberrations. Bottom, annotations of clinical, demographic and sample covariates. **e**, Number of differentially expressed features across recurrent AML alterations and proteomic assays. **f**, Pearson correlation between RNA-based AML differentiation scores and protein-level pathway ssGSEA scores. **g**, Scatter plot of ‘PI3K/AKT–mTOR’ and ‘MYC targets V2’ GSEA hallmark signatures (GSVA) at the proteome level in AML (stratified by primitive or committed classification) and bone marrow healthy control samples. *P* values were determined using a two-sided Pearson correlation test. PvC, primitive versus committed; DDR, DNA damage response; ATM, ataxia-telangiectasia mutated; EMT, epithelial mesenchymal transition; NHEJ, non-homologous end joining; ALT, alternative non-homologous end-joining; SSA, single-strand annealing. Panel **a** created in BioRender; Gondal, M. https://biorender.com/3jfo18q (2026). Source data

**Extended Data Fig. 1:**
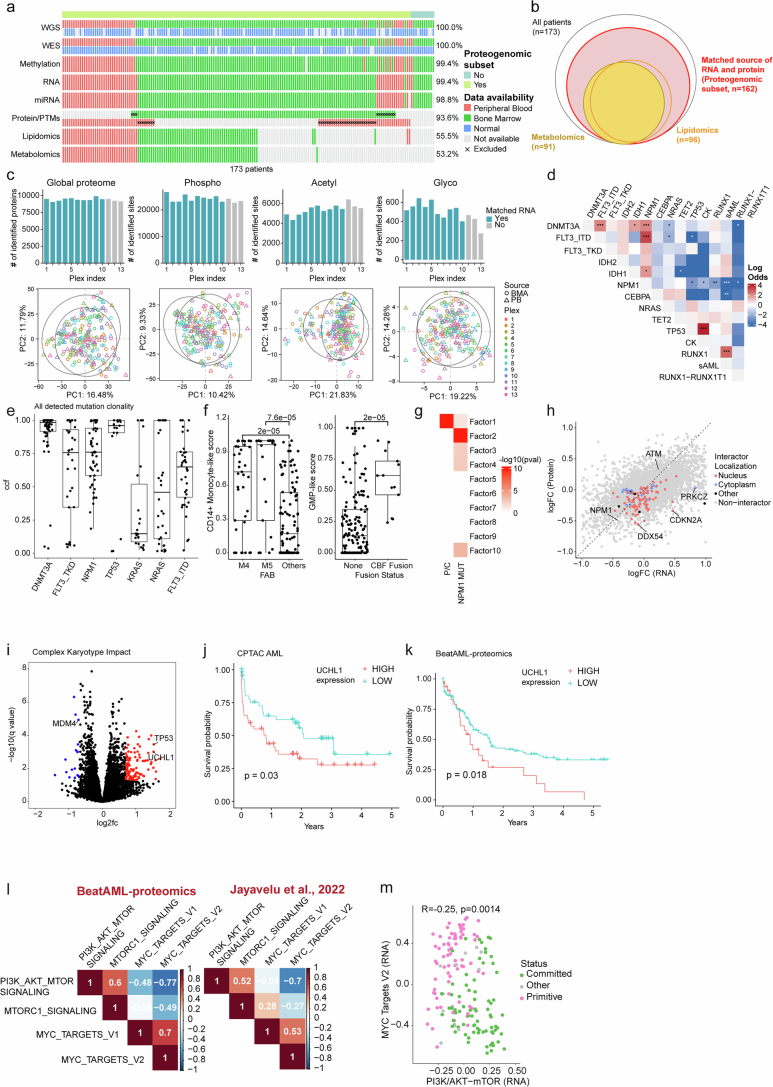
Cohort design, multi-omic data generation, and quality control. **a**. Availability of multi-omic data (DNA, RNA, protein, PTM, metabolite) types per patient. **b**. Number of patients with complete proteogenomic, metabolomic, and lipidomic data. **c**. Proteomics QC. Similar numbers of quantified proteins and PTM sites across TMT plexes (bar plots, top row), limited plex batch effects (PCA plots, bottom row). **d**. Co-occurrence and mutual exclusivity of driver genomic aberrations in AML (two sided Fisher exact test, p-values: * < 0.05, ** < 0.01, *** < 0.001). **e**. Clonality of select AML driver mutations estimated as cancer cell fraction (CCF, n = 173 patients). The box plot’s centre line represents the median, with the lower and upper boundaries indicating the first and third quartiles, and whiskers extend 1.5 times the interquartile range from the lower and upper quantiles. **f**. A significant association between CD14+ Monocyte-like scores with M4 (n = 37 patients) and M5 (n = 21 patients) French-American British classifications (FAB) AML, and granulocyte-monocyte progenitor GMP-like scores with Core Binding Factor (CBF) fusions (n = 13 patients). p-values are derived from the two-sided Wilcoxon rank-sum test. The box plot’s centre line represents the median, with the lower and upper boundaries indicating the first and third quartiles, and whiskers extend 1.5 times the interquartile range from the lower and upper quantiles. **g**. Heatmap of -log10 p-values of correlations between MOFA factor scores and primitive / committed classification (P/C) status or *NPM1* mutation status (*NPM1* MUT). **h**. Differential expression analysis at protein and RNA levels of NPM1-interacting proteins between *NPM1*-mutated and *NPM1* wild-type AML. *TP53*-mutated samples were excluded. **i**. Differential expression of proteins associated with complex karyotype status. Elevated TP53 levels due to co-enrichment with *TP53* missense mutations. **j**. Association between UCHL1 RNA expression and survival in the CPTAC cohort (median split, log-rank test). **k**. Association between UCHL1 expression and survival in the BeatAML-proteomics cohort (median split, log-rank test)a **l**. Pearson correlation patterns between MYC and MTOR pathways in two additional independent AML cohorts. **m**. Association between ‘PI3K/AKT-mTOR’ and ‘MYC Targets V2’ activity (GSVA) and primitive / committed status. p-value is calculated by the two-sided Pearson correlation test. Source data

To create a multiomic compendium of treatment-naive AML, we collected genomics, transcriptomics, proteomics, PTMs, metabolomics and lipidomics data from 173 participants (Fig. 1a and Extended Data Fig. 1a). Demographics of the participants are summarized in Supplementary Table 1. The cohort reflects the heterogeneity of AML by including major WHO subtypes across a wide age spectrum. Consistent with previous reports, participants with fusion-positive AML presented at a younger age of onset compared to those with *NPM1*-mutant or MDS-related AML (Supplementary Table 1). Complete somatic genomics (DNA, RNA-seq and miRNA-seq) for all participants, with proteomics and PTMs for 162 participants, metabolomics and lipidomics for 97 participants and matched normal DNA for 161 participants were collected (Extended Data Fig. 1b, Supplementary Table 2–6 and Methods). We detected 111,993 molecular features (Fig. 1b), with good technical reproducibility and without detrimental batch effects across tandem mass tag (TMT) plexes and specimen sources (Extended Data Fig. 1c and Methods). Tumor purity was high on average 79% (interquartile range (IQR) 73–92%). Signs of incomplete red blood cell (RBC) removal were effectively removed (Methods), together with variation associated with sample source (Supplementary Table 7 and Methods). Proteins harboring different types of PTMs were enriched in distinct cellular compartments (Fig. 1c), highlighting the unique contribution of each PTM in illuminating AML biology. We leveraged whole-genome, whole-exome and transcriptome sequencing (Methods) to detect genomic alterations including mutations, copy-number alterations, structural variants (SVs) and gene fusions (Fig. 1d and Supplementary Tables 8–13) and used those together with karyotyping, clinical covariates and central histopathological review to classify AML according to the established ELN^2^ and 2024 WHO^9^ classifications. Frequencies and patterns of co-occurrence (Extended Data Fig. 1d) across class-defining, risk-modifying and MDS-related genetic aberrations were similar to previous studies^24,25^ (Fig. 1d). Mutations in *DNTMT3A* and *TP53* were predominately clonal and mutations in *KRAS* and *NRAS* were largely subclonal; all other mutations reflected the clonal heterogeneity of AML (Extended Data Fig. 1e). Morphology-based FAB^10^ classification aligned closely with estimates of maturational phenotypes from cellular deconvolution (Extended Data Fig. 1f)^26^.

#### Distinct proteomic and post-translational impacts of class-defining AML alterations

To explore the imprint of recurrent genetic alterations on the AML proteome, we linked common genetic alterations to differences in the abundance of proteins and PTMs (Methods). We performed joint factorization of the transcriptomic and global proteomic data using multiomic factor analysis (MOFA)^27^ and found that MOFA factors 1 and 2 captured the primitive versus committed and *NPM1* mutation status (Extended Data Fig. 1g). *NPM1* mutations are known to result in aberrant cytoplasmic localization and downregulation of the NPM1 protein^28^, as observed in our data (log_2_ fold change (FC): −0.44, *P* < 0.0001). Additionally, we found that *NPM1* mutations affect the levels of hundreds of proteins (Fig. 1e), mostly suppressing known NPM1-binding partners (Extended Data Fig. 1h and Methods). Importantly, the strong imprint of *NPM1* mutations was preserved after adjusting for genetic confounders (for example, *FLT3*-ITD). Notably, CDKN2A (p16), a well-known cell-cycle regulator, tumor suppressor and nuclear NPM1 interactor, was overexpressed at the mRNA level in *NPM1*-mutant leukemias but downregulated at the protein level (Extended Data Fig. 1h).

Unlike *NPM1* mutations, the effect of WHO class-defining gene fusions was predominantly observed in PTMs and largely restricted to protein acetylation and phosphorylation (Fig. 1e). At the pathway level, the impact of genetic drivers was modest (Supplementary Tables 14–16), with notable exceptions such as *DNMT3A* mutations being linked to glycolysis, underscoring phenotypic heterogeneity with AML genetic classes^2,29^. Next, we investigated AML with complex karyotype (CK)^9^, a WHO class associated with *TP53* mutations^30^ and poor prognosis. We performed differential expression (DE) analysis and nominated UCHL1 (Extended Data Fig. 1i), a druggable deubiquitinating enzyme, as a top protein marker of CK and a significant prognostic factor in our and external AML^16^ cohorts (Extended Data Fig. 1j,k).

#### Antagonism of MYC and mTOR activity across the cellular hierarchy of AML

As cellular differentiation status is a feature of both the FAB and WHO classifications, we sought to investigate cellular heterogeneity^4,31^ and differentiation status^3,32^ in our cohort. Leveraging reference single-cell RNA-seq profiles, we deconvolved bulk profiles and inferred proportions of CD14^+^ monocytic (monocytic), GMP-like and stem-like cells (primitive)^3,4,11,31^ (Methods). We compared these differentiation scores to proteome-wide pathway activities and found significant enrichment differences (Fig. 1f). Strikingly, despite a common function in anabolism, the activity of MYC and mTOR pathways displayed opposing enrichment in our and multiple independent AML cohorts^4,33^, at both the protein (Fig. 1g and Extended Data Fig. 1l) and the RNA (Extended Data Fig. 1m) levels. Monocytic AML exhibited high mTOR signaling and low MYC activity, opposite to primitive AML.

### Integrative delineation of protein-centric AML subtypes

#### Delineation and characterization of the AML-8 protein-centric subtypes

**Fig. 2:**
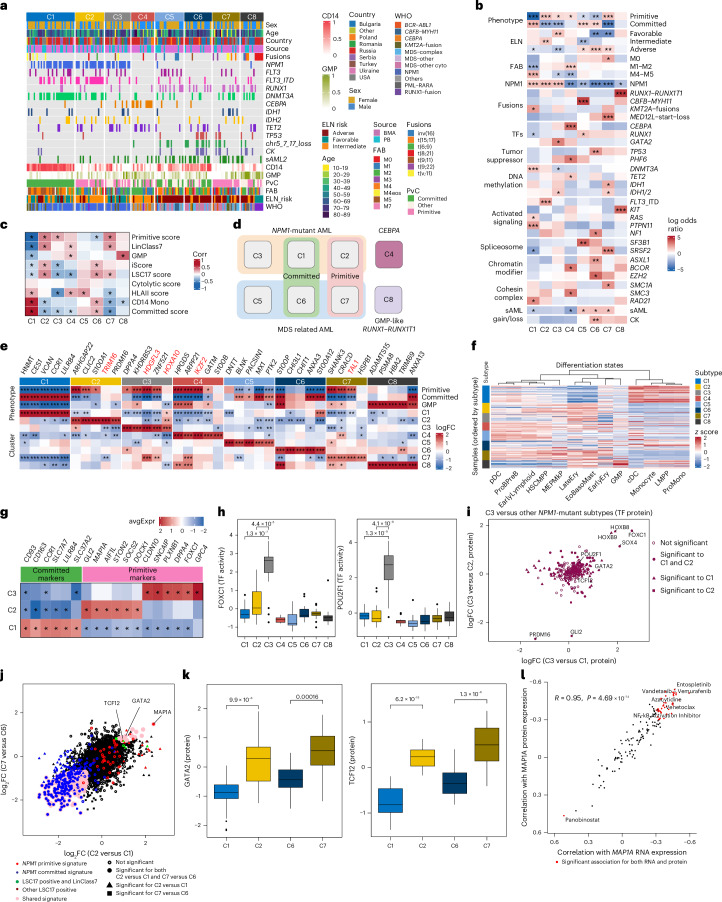
Integrative delineation of proteogenomic AML subtypes. **a**, Clinicogenomic landscape of the AML-8 subtypes identified by SNF. **b**, Statistical enrichment and mutual exclusivity of clinical attributes and genomic aberrations across the AML-8 subtypes. Asterisks indicate nominal *P* values: *P = 0.05, **P = 0.01 and ***P = 0.001, according to a two-sided Fisher exact test. **c**, Pearson correlation between AML-8 subtypes and diverse RNA-based AML differentiation scores. Asterisks indicate nominal *P* values < 0.05 according to a Pearson correlation test. **d**, Diagram summarizing the overlapping characteristics of the AML-8 subtypes in terms of enriched genetic alterations, ontogeny and differentiation. **e**, Expression of protein markers for each of the AML-8 subtypes (cluster tracks) and for AML classified according to dominant cellular differentiation (phenotype tracks). Asterisks indicate *P* values according to limma’s two-sided moderated *t*-test: *P = 0.05, **P = 0.01 and ***P = 0.001 adjusted by Benjamini–Hochberg multiple-testing correction. TFs are indicated in red. **f**, AML-8 is classified into 13 AML differentiation states using the Bone Marrow Atlas. **g**, Protein expression of selected markers of primitive and committed AML across *NPM1*-mutant subtypes. **h**, Inferred transcriptional activity (SCENIC) of FOXC1 and POU2F1 across AML-8 subtypes (*n* = 162 participants). Unadjusted *P* values were derived from a two-sided Wilcoxon rank-sum test. The box plot’s center line represents the median, with the lower and upper boundaries indicating the first and third quartiles, and whiskers extend 1.5× the IQR from the lower and upper quantiles. **i**, Differential protein expression of TFs in C3 versus other *NPM1*-mutant AML-8 subtypes (C1 and C2). **j**, Differential protein expression (log_2_FC) between primitive and committed subtypes of *NPM1*-mutant (C2 versus C1) (*x* axis) and MDS-related (C7 versus C6) (*y* axis) AML; genes from select AML transcriptional signatures are highlighted. **k**, GATA2 and TCF12 protein expression across C1 (*n* = 34 participants), C2 (*n* = 20 participants), C6 (*n* = 19 participants) and C7 (*n* = 19 participants). Unadjusted *P* values were derived from a two-sided Wilcoxon rank-sum test. The box plot statistics are the same as in **h**. **l**, Correlation of drug response (AUC) with MAP1A protein (*y* axis) and RNA expression (*x* axis) in BeatAML-proteomics. Red points represent drugs with absolute correlation values higher than 0.3 and *P* < 0.01 adjusted by Benjamini–Hochberg multiple-testing correction from a two-sided Pearson correlation test at both the RNA and the protein levels. A two-sided Pearson correlation test was also performed to report the correlation and unadjusted *P* value across *x* and *y* axes. BMA, bone marrow aspirate; PB, peripheral blood; pDC, plasmacytoid dendritic cell; ProBPreB, Pro-B and pre-B cell; HSCMPP, hematopoietic stem cell and multipotent progenitor; MEPMkP, megakaryocyte-erythroid progenitor and megakaryocyte progenitor; EoBasoMast, eosinophil, basophil and mast cell progenitor; cDC, conventional dendritic cell; LMPP, lymphoid-primed multipotent progenitor. Source data

**Extended Data Fig. 2:**
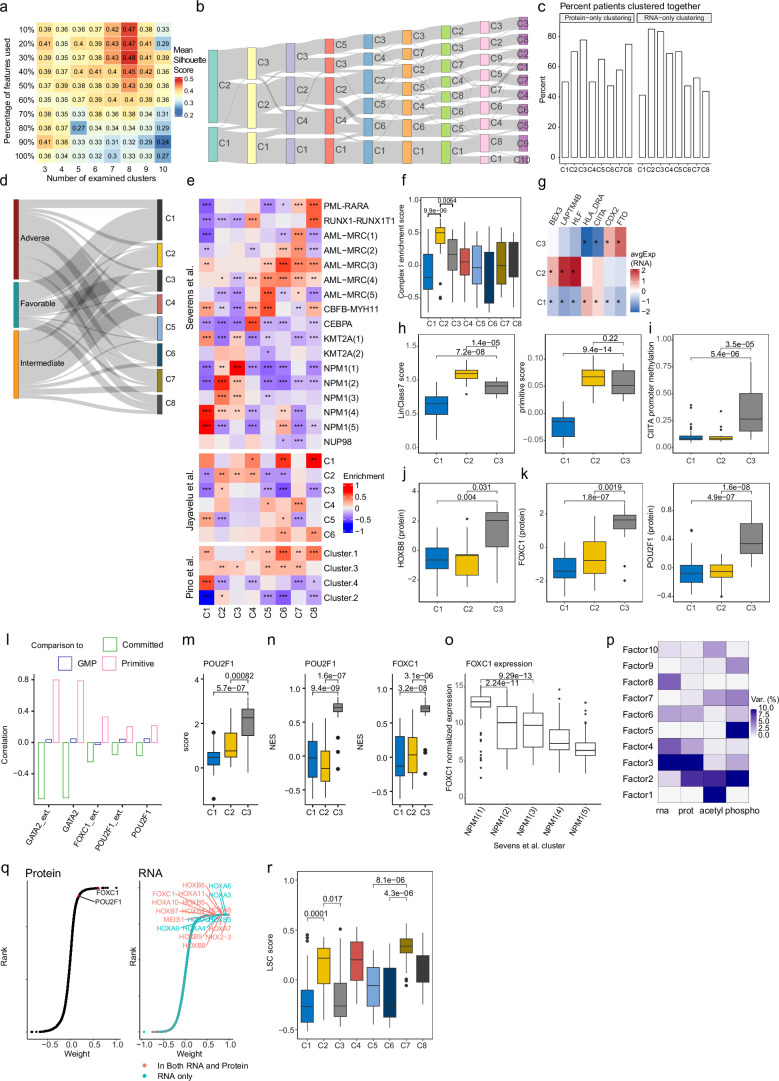
Discovery, validation, and biological interpretation of the AML-8 protein-centric subtypes. **a**. Average silhouette scores as a function of key hyperparameters for Similarity Network Fusion (SNF) clustering. **b**. Stability of SNF clustering with the number of clusters (2-10) is visualized through a Sankey diagram. **c**. Clustering concordance analysis. Percent of patients clustered together using RNA and protein who are also clustered together using RNA or protein alone. **d**. Sankey diagram showing the relationship between AML-8 and ELN classification. **e**. Enrichment of AML-8 subtype-signatures in established clusters from Jayavelu et al., Pino et al., and Severens et al. (p-values: * = 0.05, ** = 0.01, *** = 0.001). **f**. Enrichment of mitochondrial complex I proteins across AML-8 subtypes (n = 162 patients). Unadjusted p-values are derived from the two-sided Wilcoxon rank-sum test. The box plot’s centre line represents the median, with the lower and upper boundaries indicating the first and third quartiles, and whiskers extend 1.5 times the interquartile range from the lower and upper quantiles. **g**. RNA expression of select AML markers in *NPM1*-mutated AML-8 subtypes. (p-values: * = 0.05). **h**. Activity of the LinClass-7^9^ and primitive signatures^8^ in *NPM1*-mutated AML-8 subtypes (n = 162 patients). Unadjusted p-values are derived from the two-sided Wilcoxon rank-sum test. The box plot’s centre line represents the median, with the lower and upper boundaries indicating the first and third quartiles, and whiskers extend 1.5 times the interquartile range from the lower and upper quantiles. **i**. Methylation levels of the *CIITA* promoter (CpG site cg01351032) across *NPM1*-mutated AML-8 subtypes (n = 72 patients). Unadjusted p-values are derived from the two-sided Wilcoxon rank-sum test. The box plot’s centre line represents the median, with the lower and upper boundaries indicating the first and third quartiles, and whiskers extend 1.5 times the interquartile range from the lower and upper quantiles. **j**. HOXB8 protein expression across *NPM1*-mutated AML-8 subtypes (n = 72 patients). Unadjusted p-values are derived from the two-sided Wilcoxon rank-sum test. The box plot’s centre line represents the median, with the lower and upper boundaries indicating the first and third quartiles, and whiskers extend 1.5 times the interquartile range from the lower and upper quantiles. **k**. FOXC1 and POU2F1 protein expression across *NPM1*-mutated AML-8 subtypes (n = 72 patients). Unadjusted p-values are derived from the two-sided Wilcoxon rank-sum test. The box plot’s centre line represents the median, with the lower and upper boundaries indicating the first and third quartiles, and whiskers extend 1.5 times the interquartile range from the lower and upper quantiles. **l**. Correlation of activities of transcription factors from with AML differentiation. Extended targets (ext.) were included for transcription factors with fewer canonical targets. **m**. TF activity of POU2F1 using decoupleR and Dorothea (n = 72 patients). Unadjusted p-values are derived from the two-sided Wilcoxon rank-sum test. The box plot’s centre line represents the median, with the lower and upper boundaries indicating the first and third quartiles, and whiskers extend 1.5 times the interquartile range from the lower and upper quantiles. **n**. TF activity of POU2F1 and FOXC1 using SCENIC targets and GSVA (n = 72 patients). Unadjusted p-values are derived from the two-sided Wilcoxon rank-sum test. The box plot’s centre line represents the median, with the lower and upper boundaries indicating the first and third quartiles, and whiskers extend 1.5 times the interquartile range from the lower and upper quantiles. **o**. FOXC1 RNA expression in *NPM1* clusters (n = 207 patients) from Severens et al. Unadjusted p-values are derived from the two-sided Wilcoxon rank-sum test. The box plot’s centre line represents the median, with the lower and upper boundaries indicating the first and third quartiles, and whiskers extend 1.5 times the interquartile range from the lower and upper quantiles. **p**. Percentage of the variance explained by the MOFA factors across different views (rna, protein, acetylome, and phosphoproteome). **q**. Data-view specific weights for PTM-MOFA factor 4. Left: protein, Right: RNA. **r**. Venetoclax resistant leukemic stem cell (LSC) signature score across the AML-8 (n = 162 patients). Unadjusted p-values are derived from the two-sided Wilcoxon rank-sum test. The box plot’s centre line represents the median, with the lower and upper boundaries indicating the first and third quartiles, and whiskers extend 1.5 times the interquartile range from the lower and upper quantiles. Source data

To identify integrative protein-centric AML subtypes, we applied similarity network fusion (SNF) to jointly cluster the cohort’s transcriptomic and global proteomic data (Methods). We evaluated the optimal number of clusters, balancing robustness, interpretability and prognostic stratification (Extended Data Fig. 2a,b), and identified eight distinct protein-centric subtypes, henceforth referred to as the AML-8 (Fig. 2a). Both omes distinctively contribute to the integrative clustering (Extended Data Fig. 2c). Through enrichment and correlation analyses (Methods), we associated each AML-8 subtype with genetic alterations (Fig. 2b) and immunological feature scores (Fig. 2c) and established clinical classification schemes (that is, WHO, ELN and FAB; Supplementary Table 1). At the genetic level, subtypes C1–C3 are strongly enriched for *NPM1* mutations, C4 is strongly enriched for *CEBPA* mutations and C8 is strongly enriched for *RUNX1*–*RUNX1T1* t(8;21) fusions. Conversely, subtypes C5–C7 are enriched for MDS-related secondary AML (sAML) aberrations^9,34^ (Supplementary Tables 1 and 8). Notably, C8 groups genetically diverse tumors—including those with the *RUNX1*–*RUNX1T1* fusion and others with different WHO class-defining alterations—which share strikingly similar proteomic features (Fig. 2d). At the phenotypic level, we observed a separation among mature (C1 and C6), primitive (C2 and C7) and GMP-like (C8) subtypes. Additionally, each subtype was demarcated by specifically expressed protein markers (Fig. 2e), many of which are transcription factors (TFs) implicated in HSC renewal or differentiation, for example, IKZF2 in C4, TAL1 in C7 or PRDM16 in C2.

#### Comparison of AML-8 to existing clinical and phenotypic AML subtypes

To position the AML-8 classification within the context of previously published AML subtype frameworks, we performed a comparison with clinical and recent classifications. The AML-8 subtypes show clear ELN prognostic stratification, as high-risk ELN and MDS-related cases concentrate in clusters C5–C8 and C5–C7, respectively (Extended Data Fig. 2d). Conversely, favorable-risk cases are enriched in the monocytic C1 and the *NPM1*–*IDH1/2*-mutant C3 subtypes. We projected AML-8 subtypes onto a single-cell-derived framework of 13 AML differentiation states (Fig. 2f). This analysis confirmed the expected cellular identities along the axis of hematopoietic differentiation for several clusters; C1 and C6 were enriched for monocytic cell states, C8 was enriched for GMP-like cells and the primitive clusters C2 and C7 were enriched for HSC-like and multipotent progenitor-like states. We noted enrichment for early lymphoid progenitor signatures within clusters C4 and C5. We compared our classification to clusters defined by Severens et al.^35^, Jayavelu et al.^13^ and Pino et al.^16^ (Extended Data Fig. 2e). This revealed that the AML-8 framework is most similar to the transcriptomic classification by Severens et al. In contrast, the proteomics-based clusters from Jayavelu et al. appear to be more strongly driven by the axis of cellular differentiation.

#### A distinct HOXB8/9^+^ and CD99^+^*NPM1*-mutant AML subtype

We identified three distinct *NPM1*-mutated AML subtypes (C1–C3). C1 comprises mature monocytic CD14^+^ AML^31^. C2 represents primitive, triple-mutant (*NPM1*, *FLT3*-ITD and *DNMT3A*) AML and corresponds to the previously reported ‘C-mito’ subtype characterized by high mitochondrial protein abundance and sensitivity to venetoclax^13^ (Extended Data Fig. 2f). C3 has unique genetic and phenotypic characteristics compared to C1 and C2 (Extended Data Fig. 2g), with significantly fewer *DNMT3A* and *FLT3-*ITD mutations but enriched for *IDH1/2* and *GATA2* mutations (Fig. 2a,b). C2 and C3 show upregulation of distinct subsets of primitive markers (Fig. 2g and Extended Data Fig. 2h), while C3 demonstrates repression of the major histocompatiblity complex class II through *CIITA* promoter hypermethylation (Extended Data Fig. 2i). C3 is characterized by outlier expression or activity of several oncogenic TFs, as assessed by SCENIC^36^ (Supplementary Table 17–20 and Methods). The TFs most upregulated and active in C3 are *HOXB8/9*, *FOXC1*, *SOX4* and *POU2F1* (Oct1) (Fig. 2h,i and Extended Data Fig. 2j,k). Their high expression and activity are not strongly confounded by differentiation state (Extended Data Fig. 2l) and can be reproduced using alternative statistical methods (Extended Data Fig. 2m,n). Expression of *FOXC1* is also upregulated in the Severns et al. *NPM1*(1) cluster (Extended Data Fig. 2o). The multiple TFs uniquely activated in C3 (that is, *HOXB8/9* (ref. ^37^), *FOXC1* (ref. ^38^) and *SOX4* (ref. ^39^)) have been independently shown to inhibit myeloid differentiation and promote leukemias. MOFA including acetylation and phosphorylation (Extended Data Fig. 2p) identified a factor (F4) strongly linked to C4 with significant contribution from not only *FOXC1* and *POU2F1* but also a large number of HOX-family genes, *MEIS1* and *NKX2-3* (Extended Data Fig. 2q), known to be upregulated in some primitive *NPM1*-mutant tumors. Altogether, this indicates that C3 represents a distinct subset of *NPM1*-mutant AML.

#### Identification of MAP1A as a universal marker of primitive AML

The classification of AML into primitive and mature subtypes was initially proposed for *NPM1*-mutant tumors^3^, that is, C1–C3 in our cohort. However, a similar polarization is readily apparent in the three MDS-related subtypes identified here (C5–C7). Specifically, NPM1 committed signatures are upregulated in C1 (*NPM1*-mutant) and C6 (MDS-related), while *NPM1* primitive signatures together with LSC17 and LinClass7 scores are upregulated in C2 (*NPM1*-mutant) and C7 (MDS-related). This led us to investigate the differences and similarities of primitive characteristics between *NPM1*-mutant and MDS-related AML. Both primitive subtypes (C2 and C7) were equally enriched for m-LSC^40^. This observation indicates the presence in both subtypes of a subpopulation of AML cells that may develop late resistance to venetoclax^5^ (C1 and C6) (Extended Data Fig. 2r).

**Extended Data Fig. 3:**
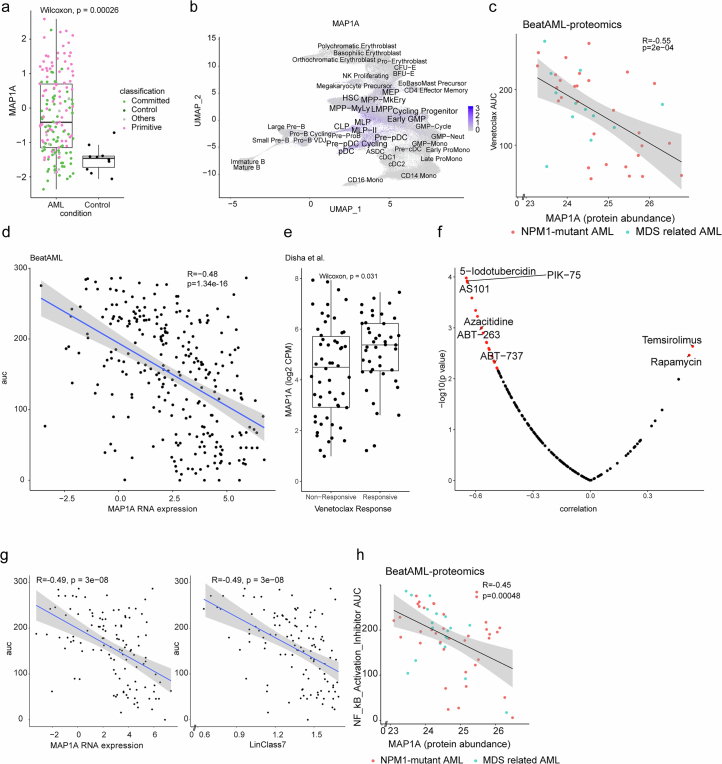
MAP1A as a universal marker of primitive AML. **a**. MAP1A protein expression in AML (n = 162 patients) and control health bone-marrow samples (n = 10 patients). p-value is derived from the two-sided Wilcoxon rank-sum test. The box plot’s centre line represents the median, with the lower and upper boundaries indicating the first and third quartiles, and whiskers extend 1.5 times the interquartile range from the lower and upper quantiles. **b**. Expression of MAP1A in a UMAP plot of myeloid and B-lineage cells from the bone marrow atlas (T-cell / NK-cell cluster omitted for space but shows no MAP1A expression). **c**. Association between MAP1A protein abundance and sensitivity (AUC) to venetoclax in the BeatAML-proteomics cohort. Confidence band (gray) is calculated from the 95% confidence level interval for predictions from the fitted linear model. p-value is calculated by the two-sided Pearson correlation test. **d**. Association of Venetoclax AUC and MAP1A RNA-expression in the non-proteomic subset of BeatAML. Confidence band (gray) is calculated from the 95% confidence level interval for predictions from the fitted linear model. p-value is calculated by the two-sided Pearson correlation test. **e**. MAP1A expression stratified by sensitivity to venetoclax from Malani et al. (n = 98 patients). p-value is derived from the two-sided Wilcoxon rank-sum test. The box plot’s centre line represents the median, with the lower and upper boundaries indicating the first and third quartiles, and whiskers extend 1.5 times the interquartile range from the lower and upper quantiles. **f**. Correlation between MAP1A RNA expression and drug AUC from Lee et al.^138^. **g**. Enrichment analysis of mitochondrial structural protein subsets (GO) with AML-8 subtypes and AML classified by differentiation. Confidence band (gray) is calculated from the 95% confidence level interval for predictions from the fitted linear model. **h**. Association between MAP1A RNA expression and LinClass7 scores with venetoclax response in BeatAML. Confidence band (gray) is calculated from the 95% confidence level interval for predictions from the fitted linear model. Source data

Next, we sought to define universal protein markers of primitive AML. We identified genes upregulated in both *NPM1*-mutant and MDS-related primitive subtypes, that is, C2 versus C1 and C7 versus C6, respectively (Fig. 2j). MAP1A, TCF12 and GATA2 exhibited higher protein expression and TF activity in C2 and C7 compared to C1 and C6 (Fig. 2k). MAP1A was also significantly higher in AML than CD34^+^ healthy bone marrow controls (Extended Data Fig. 3a). Next, to determine the cellular origin of its expression, we analyzed MAP1A at the single-cell level using the Bone Marrow Atlas^41^. This analysis revealed that MAP1A expression is high in multilymphoid progenitors and plasmacytoid dendritic cells but absent from mature hematopoietic cells of any lineage and from normal HSCs (Extended Data Fig. 3b). As primitive AML has higher response rates to venetoclax^4,33^, we evaluated the potential utility of MAP1A as an indicator of a cell state with a previously known predictive association with drug response (Fig. 2l) using the BeatAML-proteomics^16^ cohort. As hypothesized, the expression of MAP1A was strongly inversely correlated with venetoclax response (Extended Data Fig. 3c). This association was corroborated in three independent cohorts (Extended Data Fig. 3d–g). Similar results were obtained for the NF-κB inhibitor (CAS no. 545380-34-5) (Extended Data Fig. 3h). Signaling through PI3K/AKT–mTOR was suppressed in primitive AML (Fig. 1g). Together these findings demonstrate that *NPM1* mutation status and cellular differentiation principally shape the AML proteogenomic landscape and their interaction defines major subtypes.

### Landscape of metabolic reprogramming in AML

#### Parsimonious representation and intrinsic dimensionality of AML metabolism

**Extended Data Fig. 4:**
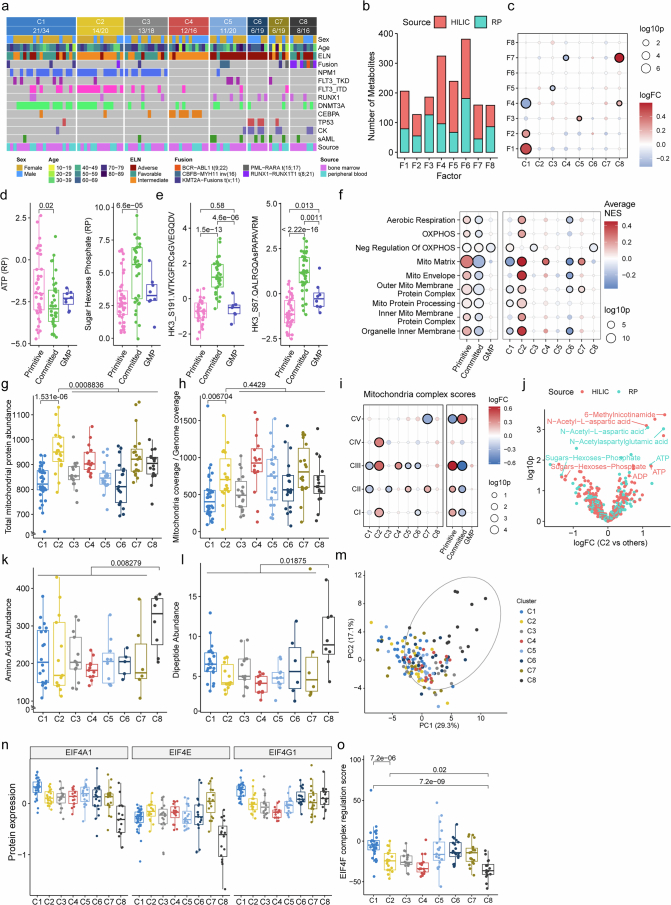
Untargeted metabolomics factorization reveals differentiation-linked metabolic states. **a**. Clinico-genomic landscape and AML-8 subtype distribution of patients with metabolomic data. **b**. Number of metabolites from each sample source associated with each ICA factor. **c**. Association of ICA factors (rows) with the AML-8 subtypes (columns). p-values calculated from limma’s two-sided moderated t-test adjusted by Benjamini-Hochberg multiple testing correction. **d**. Differential abundance of ATP (left) and sugar phosphates (right) across AML phenotypes (n = 91 patients). Unadjusted p-values are derived from a two-sided Wilcoxon rank-sum test. The box plot’s centre line represents the median, with the lower and upper boundaries indicating the first and third quartiles, and whiskers extend 1.5 times the IQR from the boundaries. **e**. Boxplot of HK3 phosphorylation at S191 and S67 sites across AML phenotypes (n = 162 patients). Unadjusted p-values are derived from a two-sided Wilcoxon rank-sum test. The box plot’s centre line represents the median, with the lower and upper boundaries indicating the first and third quartiles, and whiskers extend 1.5 times the IQR from the boundaries. **f**. Enrichment analysis of mitochondrial structural protein subsets (GO) with AML-8 subtypes and AML classified by differentiation (n = 162 patients). p-values calculated from limma’s two-sided moderated t-test adjusted by Benjamini-Hochberg multiple testing correction. **g**. Increased total mitochondrial protein abundance in C2 (primitive, n = 20 patients) relative to C1 (committed, n = 34 patients) *NPM1*-mutated AML. Unadjusted p-values are derived from a two-sided Wilcoxon rank-sum test. The box plot’s centre line represents the median, with the lower and upper boundaries indicating the first and third quartiles, and whiskers extend 1.5 times the IQR from the boundaries. **h**. Increased mitochondrial genome normalized coverage in C2 (primitive, n = 20 patients) relative to C1 (committed, n = 34 patients) and C3 *NPM1*-mutated AML. Unadjusted p-values are derived from a two-sided Wilcoxon rank-sum test. The box plot’s centre line represents the median, with the lower and upper boundaries indicating the first and third quartiles, and whiskers extend 1.5 times the IQR from the boundaries. **i**. Protein expression levels of mitochondrial complexes (CI-CV) across AML-8 subtypes and AML classified by differentiation. p-values calculated from limma’s two-sided moderated t-test adjusted by Benjamini-Hochberg multiple testing correction. **j**. Differential metabolites abundance in C2 (n = 20 patients) vs other AML-8 subtypes. p-value limma test). p-values calculated from limma’s two-sided moderated t-test adjusted by Benjamini-Hochberg multiple testing correction. **k**. Increased amino acid abundance in C8 relative to other AML-8 subtypes (n = 91 patients). Unadjusted p-values are derived from a two-sided Wilcoxon rank-sum test. The box plot’s centre line represents the median, with the lower and upper boundaries indicating the first and third quartiles, and whiskers extend 1.5 times the IQR from the boundaries. **l**. Increased dipeptide abundance in C8 relative to other AML-8 subtypes (n = 91 patients). Unadjusted p-values are derived from a two-sided Wilcoxon rank-sum test. The box plot’s centre line represents the median, with the lower and upper boundaries indicating the first and third quartiles, and whiskers extend 1.5 times the IQR from the boundaries. **m**. PCA plot based on the protein expression of EIF4F complex proteins (n = 162 patients). Ellipsis highlights location of C8 samples. **n**. Protein expression of eIF4F complex proteins EIF4A1, EIF4E, and EIF4G1 (n = 162 patients). Unadjusted p-values are derived from a two-sided Wilcoxon rank-sum test. The box plot’s centre line represents the median, with the lower and upper boundaries indicating the first and third quartiles, and whiskers extend 1.5 times the IQR from the boundaries. **o**. Boxplot showing aggregated expression score of EIF4F dependent proteins across the AML-8 (n = 162 patients). Unadjusted p-values are derived from a two-sided Wilcoxon rank-sum test. The box plot’s centre line represents the median, with the lower and upper boundaries indicating the first and third quartiles, and whiskers extend 1.5 times the IQR from the boundaries. Source data

Given the strong antagonism between MYC and mTOR (Fig. 1f,g) and their respective roles regulating cellular metabolism, we leveraged untargeted metabolomics to directly measure metabolomic differences across the AML cellular differentiation hierarchy and the AML-8 subtypes. Liquid chromatography (LC)–MS using both reverse phase (RP) LC and hydrophilic interaction liquid chromatography (HILIC) was performed (Methods) to profile 91 participant samples (Extended Data Fig. 4a and Supplementary Tables 21 and 22). After correcting for sample source and removing metabolites with high numbers of missing values (Methods), 1,780 features (putative metabolites) were detected.

**Fig. 3:**
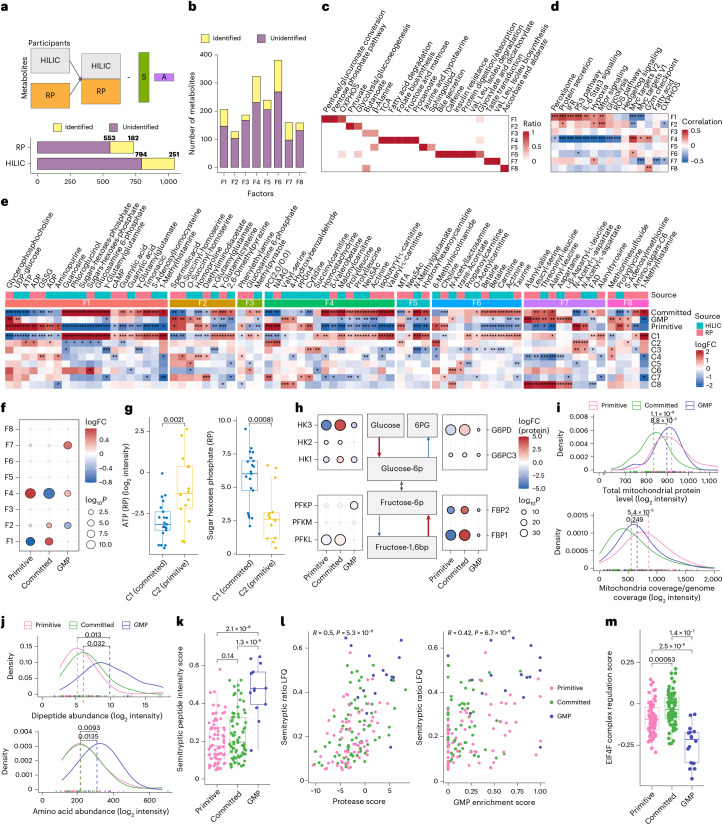
Landscape of metabolic reprogramming in AML. **a**, Top, diagram depicting metabolomics profiling of 91 participant samples using LC–MS with RP LC and HILIC. ICA factorization of the concatenated matrix into a source matrix (S) and mixing matrix (A). Bottom, number of identified and unidentified metabolites across data sources. **b**, Number of identified metabolites and unidentified spectra associated with each ICA metabolic factor. **c**, Heat map showing the overlap between metabolites in each KEGG pathway and metabolites that are positively and significantly associated with each ICA metabolic factor. **d**, Correlation between ICA metabolic factor scores and Hallmark pathways protein signature scores. *P* values were derived from a Pearson correlation test: *P = 0.05, **P = 0.01 and ***P = 0.001. **e**. Association among metabolites, phenotypes and AML-8 subtypes. Metabolites were selected on the basis of an association with phenotype or AML-8 subtype and separated on the basis of their association with ICA factors. Asterisks indicate adjusted *P* values calculated from limma’s two-sided moderated *t*-test: *P = 0.05, **P = 0.01 and ***P = 0.001 adjusted by Benjamini–Hochberg multiple-testing correction. GSSG, glutathione disulfide; cAMP, cyclic adenosine monophosphate; NAAG, *N*-acetylaspartylglutamate; FAD, flavin adenine dinucleotide. **f**, Association between ICA metabolic factors (F1–F8) and AML classified by differentiation. *P* values were calculated from limma’s two-sided moderated *t*-test adjusted by Benjamini–Hochberg multiple-testing correction. **g**, Differential abundance of ATP (left) and sugar phosphates (right) across AML-8 subtypes C1 (*n* = 20) and C2 (*n* = 13). *P* values were derived from a two-sided Wilcoxon rank-sum test. The box plot’s center line represents the median, with the lower and upper boundaries indicating the first and third quartiles, and whiskers extend 1.5× the IQR from the boundaries. **h**, Enrichment analysis of glycolysis and gluconeogenesis pathway proteins across AML classified by differentiation. 6PG, 6-phosphogluconate. *P* values were calculated from limma’s two-sided moderated *t*-test adjusted by Benjamini–Hochberg multiple-testing correction. **i**, Total mitochondrial protein abundance (top) and normalized mitochondrial DNA coverage (bottom) (*n* = 91 participants). Unadjusted *P* values were derived from a two-sided Wilcoxon rank-sum test. The vertical line indicates the median of samples. **j**. Abundance of amino acids and dipeptides across AML classified by differentiation (*n* = 91 participants). Unadjusted *P* values were derived from a two-sided Wilcoxon rank-sum test. The vertical line indicates the median of samples. **k**, Increased relative abundance of semitryptic peptides in GMP-like AML (C8; *n* = 16 participants). Unadjusted *P* values were derived from a two-sided Wilcoxon rank-sum test. The box plot’s center line represents the median, with the lower and upper boundaries indicating the first and third quartiles, and whiskers extend 1.5× the IQR from the boundaries. **l**, Correlation of protease expression (left) and GMP differentiation (right) scores with semitryptic peptide abundance across AML classified by differentiation. Unadjusted *P* values were derived from a two-sided Pearson correlation test. **m**, Aggregated expression score of EIF4F-dependent proteins across AML classified by differentiation (*n* = 162 participants). Unadjusted *P* values were derived from a two-sided Wilcoxon rank-sum test. The box plot’s center line represents the median, with the lower and upper boundaries indicating the first and third quartiles, and whiskers extend 1.5× the IQR from the boundaries. Source data

Of the 1,780 detected features, 433 metabolites (322 unique) could be identified at varying levels of confidence (Supplementary Tables 21 and 22), while 1,347 remained unidentifiable despite the use of multiple spectral libraries (Fig. 3a and Methods). To overcome this, we applied independent component analysis (ICA) (Methods, Fig. 3a and Supplementary Tables 23 and 24) to identify latent factors comprising both identified and unidentified metabolites (Fig. 3b and Extended Data Fig. 4b). By contrasting the goodness of fit with model complexity (Methods), we selected eight metabolomic factors (F1–F8). To biologically interpret these factors, we associated them with Kyoto Encyclopedia of Genes and Genomes (KEGG) metabolic pathways, either directly through the intersection of metabolites with positive and significant scores (that is, loadings) for factors with KEGG pathways (Fig. 3c) or indirectly by correlating the activity of metabolic pathways with factor scores across samples (Fig. 3d). Notably, F1 was associated with mTOR, while F4 was associated with MYC activity, demonstrating at the metabolomic level the dichotomized effects of these two pathways.

#### Metabolomic shift between primitive and committed AML

We performed enrichment analysis to determine whether the AML-8 protein-centric subtypes (Extended Data Fig. 4c) or AML classified by differentiation state had characteristic metabolic profiles (Fig. 3e,f). We found that F1 and F4 comprise metabolites with large differences in abundance between primitive and committed AML (Fig. 3e), reflecting a striking metabolic shift between committed AML associated with F1 and primitive AML associated with F4 (Fig. 3f). Metabolic changes contributing to F1 include upregulation of sugar phosphates, such as sugar-hexose-phosphate, and downregulation of nucleoside phosphates, such as ATP (Extended Data Fig. 4d), most exemplified with the committed C1 and primitive C2 comparison (Fig. 3g). The observed differences in energy metabolism are consistent with the upregulation of key glycolysis pathway enzymes HK1, HK2, HK3, FBP1 and FBP2 at the protein level (Fig. 3h) and increased phosphorylation of HK3 (Extended Data Fig. 4e).

One recent metabolic finding indicated increased reliance of leukemic stem cells on oxidative phosphorylation (OXPHOS)^42^, as opposed to normal HSCs, which are glycolytic^43^. We interrogated primitive AML to determine whether the observed increases in OXPHOS signature and ATP production (Fig. 3g and Extended Data Fig. 4d) were associated with mitochondrial abundance as OXPHOS processes take place in mitochondria. We found that primitive AML samples had significantly more mitochondrial proteins and mitochondrial DNA than committed AML (Fig. 3i). Further examination revealed an increased abundance of multiple mitochondrial protein subsets in primitive AML samples, which was especially prominent in the primitive *NPM1*-mutant subtype C2 (Extended Data Fig. 4f). Compared to C1, the committed *NPM1*-mutant subtype C2 had an increased abundance of mitochondrial proteins, more copies of the mitochondrial genome, higher expression of mitochondrial complex proteins and increased ATP production (Extended Data Fig. 4g–j).

#### Catabolic activity results in amino acids and dipeptide accumulation in GMP

We observed one metabolic factor F7 to be highly specific to C8 (Fig. 3e,f). Metabolites positively correlated with this factor consisted of amino acids and dipeptides including prolyl-alanine, lysyl-leucine and histidyl-alanine (Supplementary Table 25). We found a significantly higher abundance of amino acids and dipeptides in GMP-like AML (Fig. 3j and Extended Data Fig. 4k,l). Semitryptic peptides are direct products of protein degradation^44^. We measured their abundance through custom proteomics searches (Methods) and found that GMP-like AML has significantly elevated semitryptic peptide levels (Fig. 3k). The accumulation of amino acids, dipeptides and semitryptic peptides in GMP-like AML suggests an imbalance between protein degradation and synthesis, prompting us to explore the dysregulation of proteases and/or the translation machinery^15^. We found both elevated levels of multiple proteases including ELANE and PRSS57, relative to both primitive and committed AML (*P* < 0.01), and a striking loss of EIF4E, a key member of the eukaryotic translation initiation factor complex eIF4F^45^ (Extended Data Fig. 4m,n). We found that an aggregate protease score was more strongly correlated with semitryptic peptide abundance than the degree of GMP differentiation (Fig. 3l and Methods). Accordingly, we observed a significant downregulation of proteins that depend on eIF4F for translation (Fig. 3m, Extended Data Fig. 4o and Methods). Notably, inhibition of amino acid metabolism is an emerging strategy to target leukemic stem cells^46^.

### Lipidome remodeling across the AML cellular differentiation hierarchy

#### Patterns of lipid coregulation within and across lipid subclasses and AML subtypes

**Fig. 4:**
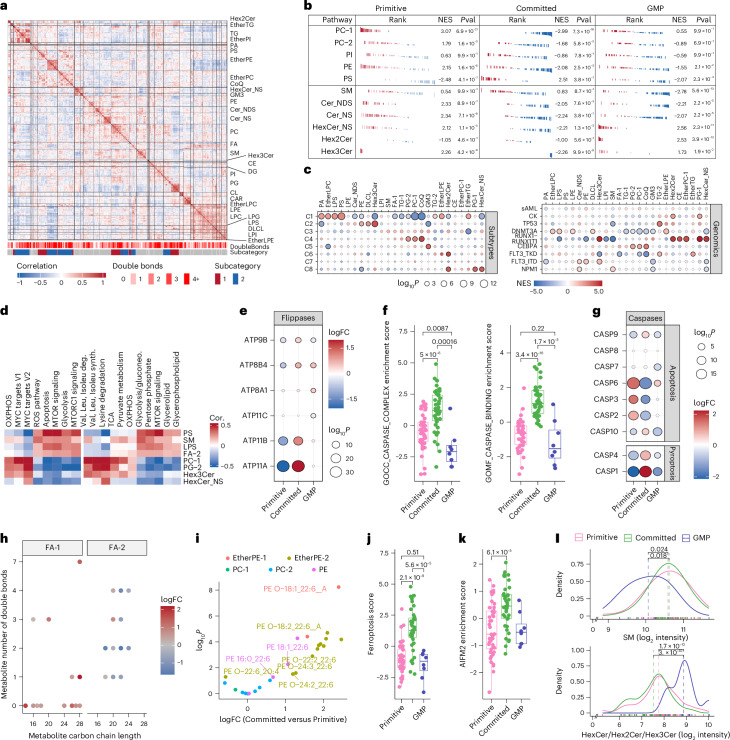
Lipidome remodeling across the AML cellular differentiation hierarchy. **a**, Heat map of lipid–lipid correlations. Lipids are arranged by lipid ontology class (blocks) and further hierarchically clustered within each block. Pearson correlation was performed. TG, triglyceride; PA, phosphatidic acid; PE, phosphatidylethanolamine; CoQ, coenzyme Q; PC, phosphatidylcholines; DG, diglyceride; CL, cardiolipin; CAR, acetylcarnitine; LPE, lysophosphatidylethanolamine; LPC, lysophosphatidylcholines; DLCL, dilysocardiolipin; LPI, pysophosphatidylinositol; NS, ceramide; NS NDS, ceramide NDS. **b**, Association (differential GSEA enrichment) of lipid ontology classes with AML classified by differentiation visualized through a barcode plot. *P* values were derived from a permutation test. **c**, Association of lipid ontology classes with the AML-8 subtypes and select driver genomic aberrations. *P* values were calculated from limma’s two-sided moderated *t*-test adjusted by Benjamini–Hochberg method. **d**, Correlation of lipid class enrichment among Hallmark (left) and KEGG protein pathways (right). Pearson correlation was performed. **e**, Dot plot showing decreased flippase enrichment in primitive AML. *P* values were calculated from limma’s two-sided moderated *t*-test adjusted by Benjamini–Hochberg multiple-testing correction. **f**, Box plot of caspase complex signatures across AML samples (*n* = 162 participants). *P* values were derived from a two-sided Wilcoxon rank-sum test. The box plot’s center line represents the median, with the lower and upper boundaries indicating the first and third quartiles, and whiskers extend 1.5× the IQR from the boundaries. **g**, Dot plot showing the association of caspase proteins with phenotypes. *P* values were calculated from limma’s two-sided moderated *t*-test adjusted by Benjamini–Hochberg. **h**, Increased enrichment of FA-1 and decreased FA-2 in primitive compared to committed AML samples. Individual lipids were plotted against carbon chain length and number of double bonds. **i**, Scatter plot showing increased 22:6 lipids in committed AML. *P* values were calculated from limma’s two-sided moderated *t*-test adjusted by Benjamini–Hochberg multiple-testing correction. **j**, Box plot of ferroptosis signature across AML (*n* = 96 participants). Unadjusted *P* values were derived from a two-sided Wilcoxon rank-sum test. The box plot’s center line represents the median, with the lower and upper boundaries indicating the first and third quartiles, and whiskers extend 1.5× the IQR from the boundaries. **k**, Box plot of AIFM2 protein expression across AML (*n* = 162 participants). Unadjusted *P* values were derived from a two-sided Wilcoxon rank-sum test. The box plot’s center line represents the median, with the lower and upper boundaries indicating the first and third quartiles, and whiskers extend 1.5× the IQR from the boundaries. **l**, Density plot showing increased abundance of HexCer and decreased SM abundance in GMP-like AML (*n* = 96 participants). *P* values were derived from a two-sided Wilcoxon rank-sum test. The vertical line indicates the median of samples. Source data

**Extended Data Fig. 5:**
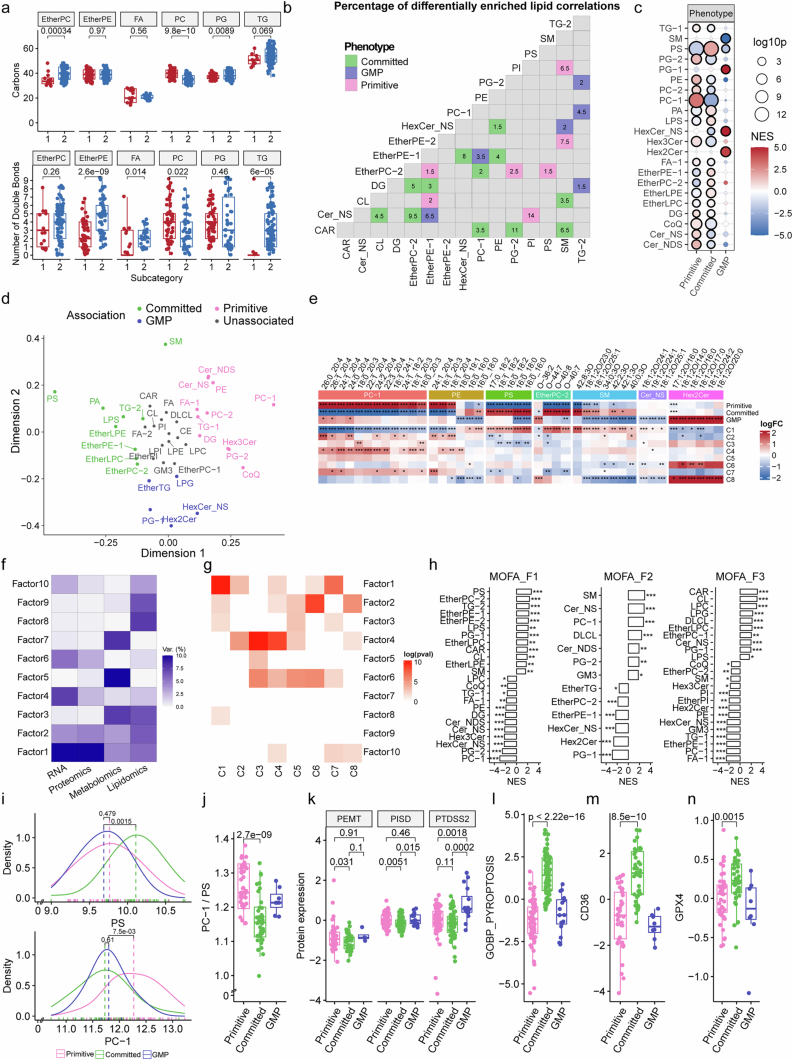
Lipidome remodeling across AML differentiation. **a**. Comparisons of carbon length and number of double bonds between each subcategory of EtherPC, EtherPE, FA, PC, PG, and TG. (EtherPC-1 n = 14 metabolites, EtherPC-2 n = 90 metabolites, EtherPE-1 n = 58 metabolites, EtherPE-2 n = 67 metabolites, FA-1 n = 15 metabolites, FA-2 n = 29 metabolites, PC-1 n = 62 metabolites, PC-2 n = 51 metabolites, PG-1 n = 55 metabolites, PG-2 n = 45 metabolites, TG-1 n = 10 metabolites, TG-2 n = 96 metabolites). Unadjusted p-values are derived from a two-sided Wilcoxon rank-sum test. The box plot’s centre line represents the median, with the lower and upper boundaries indicating the first and third quartiles, and whiskers extend 1.5 times the IQR from the boundaries. **b**. Percentage of significant correlations between classes of lipids across phenotypes. **c**. Association (single-sample GSEA enrichment) of lipid ontology classes with AML classified by differentiation visualized through a bubble plot. p-values calculated from limma’s two-sided moderated t-test adjusted by Benjamini-Hochberg multiple testing correction. **d**. Association (correlation) of lipid ontology classes with AML classified by differentiation visualized through a 2D PCA plot. Each lipid class is assigned to the most positively correlated differentiation state. **e**. Overview of individual lipid enrichments in AML-8 subtypes and phenotypes. p-values calculated from limma’s two-sided moderated t-test adjusted by Benjamini-Hochberg multiple testing correction: * = 0.05, ** = 0.01, *** = 0.001. **f**. Percentage of the variance explained by the MOFA factors across different views (RNA, protein, metabolomics, and lipidomics). **g**. Association between MOFA factors and AML-8 subtypes. Two-sided Pearson correlation test is applied and p-values adjusted by Benjamini-Hochberg. **h**. Correlation between MOFA factors F1-F3 and lipid classes. p-values are derived from a permutation test: * < 0.05, ** < 0.01, *** < 0.001. **i**. Density plot showing decreased abundance of PS and increased abundance of PC-1 for primitive compared to committed phenotype(n = 96 patients). Unadjusted p-values are derived from the two-sided Wilcoxon rank-sum test. The vertical line indicates the median of samples. **j**. Boxplot showing increased PC-1/PS ratio in primitive AML(n = 96 patients). Unadjusted p-values are derived from a two-sided Wilcoxon rank-sum test. The box plot’s centre line represents the median, with the lower and upper boundaries indicating the first and third quartiles, and whiskers extend 1.5 times the IQR from the boundaries. **k**. Boxplot showing increased expression of PC conversion related proteins (PEMT, PISD, and PTDSS2) in primitive compared to committed AML(n = 96 patients). Unadjusted p-values are derived from a two-sided Wilcoxon rank-sum test. The box plot’s centre line represents the median, with the lower and upper boundaries indicating the first and third quartiles, and whiskers extend 1.5 times the IQR from the boundaries. **l**. Boxplot showing decreased GOBP pyroptosis signature scores in primitive compared to committed AML (n = 96 patients). Unadjusted p-values are derived from a two-sided Wilcoxon rank-sum test. The box plot’s centre line represents the median, with the lower and upper boundaries indicating the first and third quartiles, and whiskers extend 1.5 times the IQR from the boundaries. **m**. Boxplot showing increased CD36 protein expression in committed AML (n = 96 patients). Unadjusted p-values are derived from a two-sided Wilcoxon rank-sum test. The box plot’s centre line represents the median, with the lower and upper boundaries indicating the first and third quartiles, and whiskers extend 1.5 times the IQR from the boundaries. **n**. Boxplot showing increased GPX4 protein expression in committed AML (n = 96 patients). Unadjusted p-values are derived from a two-sided Wilcoxon rank-sum test. The box plot’s centre line represents the median, with the lower and upper boundaries indicating the first and third quartiles, and whiskers extend 1.5 times the IQR from the boundaries. Source data

As AML cells showed distinct metabolic profiles depending on their differentiation stage, we investigated whether these differences extended to the global lipidome. Using LC–MS-based lipidomics, we detected 1,365 lipids across 96 participant samples (Supplementary Tables 26–28 and Methods). Lipids within a subclass were strongly correlated (Fig. 4a), enabling robust analysis at the ontological level (length and unsaturation)^47^ (for example, ether-linked phosphatidylcholines; Extended Data Fig. 5a and Methods). To characterize differences in lipid–lipid coregulation across AML maturation states, we calculated differential lipid–lipid correlations (Methods) and identified distinct coregulatory patterns in primitive, committed and GMP-like AML (Extended Data Fig. 5b).

Analysis of lipid abundances across AML classified by differentiation revealed distinct associations. Phosphatidylserine (PS) and sphingomyelin (SM) were enriched in committed cells, while phosphatidylcholine (PC) was elevated in primitive cells. Elevated hexosylceramides and dihexosylceramides (HexCer and Hex2Cer) were characteristic of GMP-type AML cells (Extended Data Fig. 5c,d and Supplementary Table 29). These associations extended to other major diacylphospholipids, such as phosphatidylinositol (PI) and phosphatidylethanolamine (PE), as well as ceramide (Cer), which is at the center of sphingolipid metabolism (Fig. 4b). Analysis of lipid abundance at both the protein-centric subtype and genetic levels (Fig. 4c and Extended Data Fig. 5e) revealed that differences in lipid profiles were primarily associated with the degree of cellular differentiation, rather than specific AML-8 subtypes or driver mutations. Joint factorization of RNA, protein, metabolomic and lipidomic data (METABO-MOFA) found that MOFA factors F1 (ether lipids) and F3 (fatty acids, FAs) distinguished primitive from committed AMLs, while F2 (SMs and Cers) defined a GMP-like axis (Extended Data Fig. 5f–h). These observations were supported by correlations between lipid abundance and functional pathways (Fig. 4d).

#### Lipidome signatures of cell death mechanisms in AML

PS lipids have been implicated in the control of AML stemness^48^ and immunosuppression^49^. In primitive AML, we observed PS depletion and accumulation of PC-1 (that is, PCs with polyunsaturated FAs (PUFAs)) resulting in a higher PC-1/PS ratio (Extended Data Fig. 5i,j). PC-related biosynthetic proteins were also upregulated (Extended Data Fig. 5k), while flippases ATP11A and ATP11B were decreased (Fig. 4e). Committed AML, however, had opposing signatures of increased PS and flippase activity, which leads to the externalization of PS to the cell surface, inducing cell death through caspase activation. Accordingly, we observed reduced caspase activity in primitive and elevated caspase activity in committed AML (Fig. 4f). This points to antiapoptotic mechanisms in primitive AML, while committed AML displays proapoptotic signatures, enriched for pyroptosis markers CASP1 and CASP4 (Fig. 4g and Extended Data Fig. 5l).

Committed AML samples were enriched in unsaturated FA-2 over saturated FA-1 (Fig. 4h). These differences may be attributable to lipid uptake by the FA transporter CD36 protein, which is highly expressed in committed AML (Extended Data Fig. 5m). We observed an elevation of peroxidizable ether phospholipids containing the PUFA 22:6 (Fig. 4i) and signatures of ferroptosis in committed AML (Fig. 4j). Ferroptosis can be counteracted by the proteins AIFM2 (also known as ferroptosis suppressor protein 1) (Fig. 4k) or GPX4 (Extended Data Fig. 5n), both of which were increased in committed AML. Our result suggests modulation of ferroptosis may be a druggable pathway for committed AML.

#### HexCer and Cer salvage pathway elevated in GMP-like AML

Finally, our analysis revealed a distinctive relationship between GMP-like AML and sphingolipid metabolism, characterized by an increase in HexCer levels and a concurrent depletion of SM (Fig. 4l and Extended Data Fig. 5c). As overall Cer levels were not increased (nor was de novo synthesis), Cers were potentially used, contributing to the accumulation of HexCer lipids (Fig. 4l and Extended Data Fig. 5c). Overall, lipidomics helped to contextualize molecular differences and outcomes in primitive, committed and GMP-like AML.

### Genetic and metabolic control of protein acetylation and epigenetic remodeling

#### Revealing global changes in protein acetylation levels

**Fig. 5:**
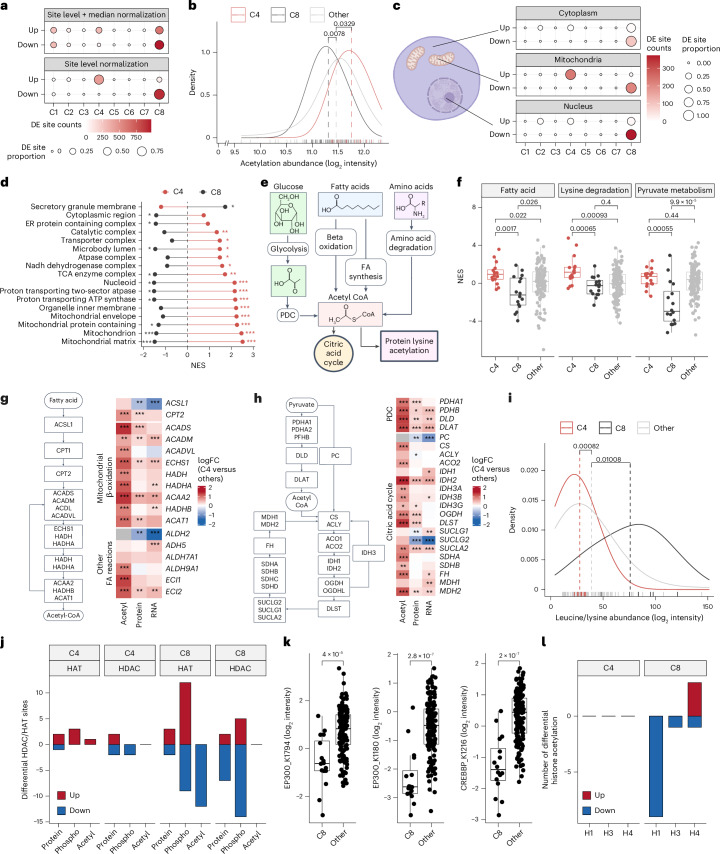
Genetic and metabolic control of protein acetylation and epigenetic remodeling. **a**, Proportion and number of differential acetylation sites in each subtype identified using different normalization methods. **b**, Acetylation abundance differences between C4 (*n* = 16 participants), C8 (*n* = 16 participants) and other subtypes. *P* values were derived from a two-sided Wilcoxon rank-sum test. The vertical line indicates the median of samples. **c**, Proportion of differential acetylation sites separated by protein localization in each subtype. **d**, Enrichment of pathways in which genes are differentially hyperacetylated or hypoacetylated in C4 (*n* = 16 participants) and C8 (*n* = 16 participants). *P* values were derived from a permutation test: *P = 0.05, **P = 0.01 and ***P = 0.001. **e**, Diagram of acetyl-CoA-producing pathways. **f**, Enrichment of protein pathways related to acetyl-CoA synthesis and degradation (*n* = 162 participants). Unadjusted *P* values were derived from a two-sided Wilcoxon rank-sum test. The box plot’s center line represents the median, with the lower and upper boundaries indicating the first and third quartiles, and whiskers extend 1.5× the IQR from the boundaries. **g**, Diagram of FA degradation enzymes (KEGG:hsa00071, left) with corresponding heat map (right) of differential acetyl, protein and gene expression between C4 and other subtypes. *P* values were calculated from limma’s two-sided moderated *t*-test adjusted by Benjamini–Hochberg multiple-testing correction. **h**, Diagram of TCA cycle enzymes (KEGG:hsa00020, left) with corresponding heat map (right) of differential acetyl, protein and gene expression between C4 and other subtypes. *P* values were calculated from limma’s two-sided moderated *t*-test adjusted by Benjamini–Hochberg. **i**, The abundance of amino acids leucine and lysine in C4 (*n* = 16 participants), C8 (*n* = 16 participants) and other subtypes. *P* values were derived from a two-sided Wilcoxon rank-sum test. The vertical line indicates the median of samples. **j**, Upregulation and downregulation of HDAC and HAT sites in C4 and C8 subtypes. **k**, Abundance of acetylation sites EP300 K1794, EP300 K1180 and CREBBP K1216 across AML (*n* = 162 participants). Unadjusted *P* values were derived from a two-sided Wilcoxon rank-sum test. The box plot’s center line represents the median, with the lower and upper boundaries indicating the first and third quartiles, and whiskers extend 1.5× the IQR from the boundaries. **l**, Number of differentially expressed histone acetylation sites. Panel **c** partially created in BioRender; Gondal, M. https://biorender.com/uy7vuos (2026). Source data

**Extended Data Fig. 6:**
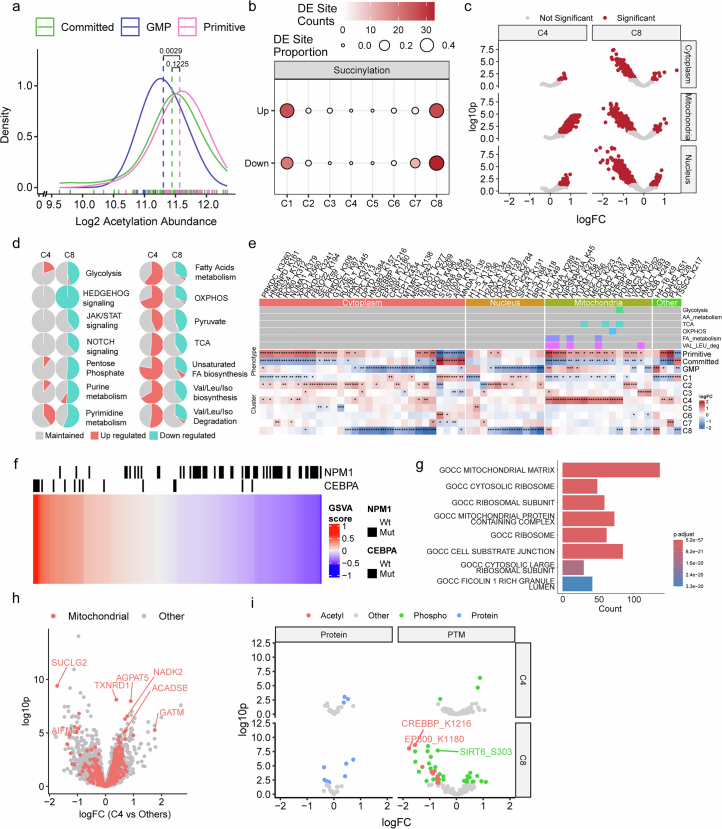
Protein acetylation remodeling in AML. **a**. Acetylation abundance across phenotypes (n = 162 patients). Unadjusted p-values are derived from the two-sided Wilcoxon rank-sum test. The vertical line indicates the median of samples. **b**. Volcano plots showing significant acetylation sites for C4 and C8 separated by localization of genes. **c**. Dotplot showing the number of differential succinylation sites across AML-8 subtypes. p-values calculated from limma’s two-sided moderated t-test adjusted by Benjamini-Hochberg. **d**. Pie charts depicting the number of differential acetylation sites within each protein pathway for C4 (n = 16 patients) and C8 (n = 16 patients). **e**. Heatmap showing significant acetylation sites identified for each subtype. p-values calculated from limma’s two-sided moderated t-test, adjusted by Benjamini-Hochberg. Asterisk indicate p-value: * < 0.05, ** < 0.01, *** < 0.001. **f**. Heatmap of C4 subtype-specific protein signature applied to Jayavelu et al. **g**. Overrepresentation analysis of acetylated proteins in MOFA factor 1. Adjusted p-values are derived from the permutation test. **h**. Differentially expressed proteins in C4 (n = 16 patients) vs others. Mitochondrially related genes are highlighted. p-values calculated from limma’s two-sided moderated t-test adjusted by Benjamini-Hochberg multiple testing correction. **i**. Volcano plots showing differential protein and PTM related to HDAC or HATs in C4 (n = 16 patients) and C8 (n = 16 patients) compared to other subtypes. p-values calculated from limma’s two-sided moderated t-test adjusted by Benjamini-Hochberg multiple testing correction. Source data

Changes in metabolism are often accompanied by changes in acetyl-CoA, a key metabolite in protein acetylation and energy production. Median centering is typically performed to normalize PTM signals across samples. However, this can also result in spurious results when global differences are incorrectly removed^50^ (Fig. 5a and Methods). Therefore, we analyzed the non-median-centered acetylation data and found striking hyperacetylation in C4 and hypoacetylation in C8 (Fig. 5b). These global changes were neither fully explained by a differentiation-based classification of AML (Extended Data Fig. 6a) nor extended to the related PTM succinylation (Extended Data Fig. 6b) and could be replicated by restricting the analysis to just *CEBPA*-mutant AMLs. Next, we stratified acetylation sites by the primary cellular localization of their target proteins and found that, while hyperacetylation in C4 occurred almost exclusively within the mitochondria, C8 hypoacetylation occurred across all cellular compartments (Fig. 5c and Extended Data Fig. 6c–e). The difference was further reflected in cellular pathways, with C4 hyperacetylation impacting multiple mitochondrial energy pathways such as tricarboxylic acid (TCA) cycle, pyruvate cycle and OXPHOS (Fig. 5d). Integrative factor analysis (PTM-MOFA) identified a latent factor (F1) driven predominantly by acetylation data (Extended Data Fig. 6f) and strongly associated with the C4 subtype; subsequent analysis traced this factor to the hyperacetylation of mitochondrial proteins (Extended Data Fig. 6g).

#### Nonenzymatic hyperacetylation of mitochondrial proteins in *CEBPA*-mutant AML (C4)

Hyperacetylation in the mitochondria is a nonenzymatic process involving acetyl-CoA^51^; however, as the measurement of acetyl-CoA is typically not amenable to untargeted metabolomics analyses^52^, we investigated the pathways that generate acetyl-CoA: pyruvate dehydrogenase, β-oxidation of FAs and degradation of amino acids (Fig. 5e). Analysis of these pathways revealed that, in the C4 subtype, FA and lysine degradation pathways exhibited significantly higher activity, while pyruvate metabolism remained unchanged (Fig. 5f). To dissect this further, we analyzed FCs in protein, RNA and acetylation levels within two key pathways: FA β-oxidation (Fig. 5g) and the TCA cycle (Fig. 5h). In C4, FA β-oxidation showed enriched protein expression and acetylation, indicating acetyl-CoA accumulation. Most TCA cycle and mitochondrial genes were concurrently upregulated, with the notable exception of *SUCLG2* (Extended Data Fig. 6h). SUCLG2 activity inhibits the TCA cycle, causing a reversal of flux and resulting in increased acetyl-CoA levels^53^. Enriched valine, leucine and isoleucine degradation pathways, combined with leucine and lysine depletion, further contributed to acetyl-CoA accumulation (Fig. 5i).

#### Global hypoacetylation and PTM dysregulation of acetyl writers and erasers in GMP-like AML (C8)

Unlike C4’s mitochondria-restricted hyperacetylation, C8 displayed broad hypoacetylation across multiple compartments (Fig. 5c). Despite stable histone deacetylase (HDAC) and histone acetyltransferase (HAT) protein levels, C8 uniquely exhibited drastic phosphorylation and acetylation changes on these regulators, suggesting a PTM-driven regulatory mechanism (Fig. 5j and Extended Data Fig. 6i). These include significant deacetylation at EP300 K1794, important for EP300 HAT activity^54^, and commonly acetylated sites such as EP300 K1180 and CREBBP K1216 according to PhosphoSitePlus^55^ (Fig. 5k). The dysregulation of HATs and HDACs, enzymes that directly acetylate and deacetylate histone tails, respectively, was further reflected at the PTM level in the number of differentially acetylated histone sites (Fig. 5l), supporting a functional regulatory role of these PTMs.

### Functional network analysis prioritizes MTA1 as a mediator of HDAC inhibitor resistance

**Fig. 6:**
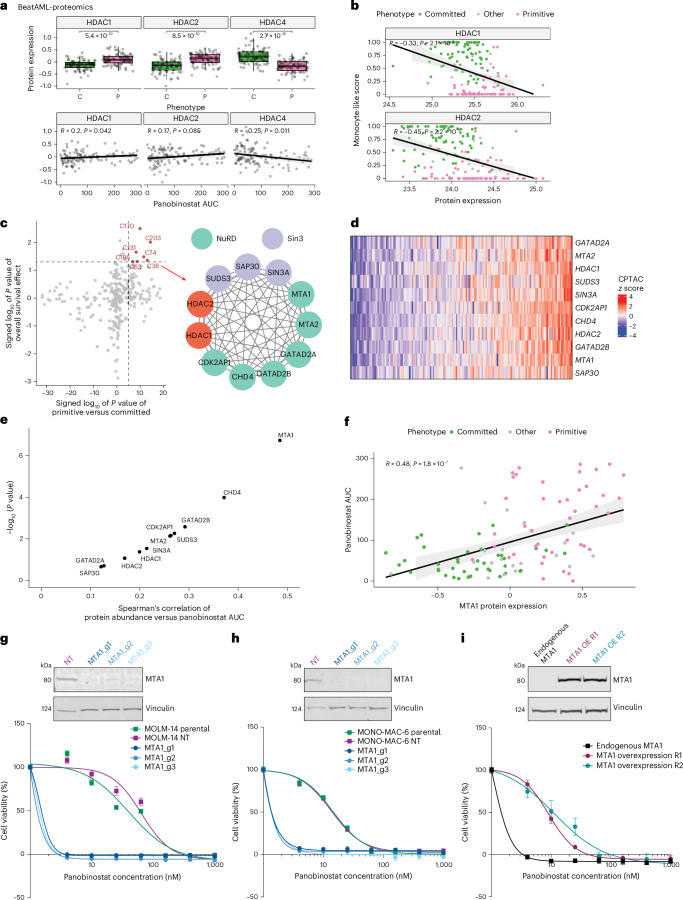
Cofunctional network analysis using FunMap reveals MTA1 as a mechanism of panobinostat resistance. **a**, Protein expressions of HDAC1, HDAC2 and HDAC4 in primitive (P; *n* = 74) and committed (C; *n* = 74) samples and their correlations with panobinostat AUC values in BeatAML-proteomics^16^ cohort. Unadjusted *P* values of box plots were derived from a two-tailed Student *t*-test. Boxes represent the IQR and whiskers extend to 1.5× the IQR or to the minimum and maximum values if no point exceeds that range. No outliers were removed from the plots. Spearman rank correlation coefficients and unadjusted two-tailed *P* values are shown. **b**, Scatter plot of protein abundance of HDAC1/2 and monocyte-like score. Spearman rank correlation coefficients and unadjusted two-tailed *P* values are shown. **c**, Difference in clique activity between primitive and committed samples and their associations with overall survival. A total of 11 genes in clique C38 are highlighted on the right. Unadjusted *P* values of box plots were derived from a two-tailed Student *t*-test. **d**, Distribution of protein abundance of 11 genes within the C38 clique across this cohort. **e**, Spearman correlation between the protein abundance of 11 C38 genes and the AUC values of panobinostat in the BeatAML-proteomics cohort. **f**, Correlation of MTA1 protein expression with panobinostat AUC in the BeatAML-proteomics cohort. Spearman rank correlation coefficients and unadjusted two-tailed *P* values are shown. **g**, Knockout of *MTA1* restores sensitivity to panobinostat in the MOLM-14 cell line (bottom; *n* = 2), with western blot confirming successful knockout of MTA1 (top). NT, nontargeting control. **h**, Knockout of *MTA1* restores sensitivity to panobinostat in MONO-MAC-6 cell line (bottom; *n* = 2), with western blot confirming successful knockout of MTA1. **i**, MTA1 overexpression (OE) promotes resistance against panobinostat in a quizartinib-resistant MOLM-14 cell line (bottom; *n* = 2), with western blot confirming successful overexpression of MTA1 (top). Error bars represent the s.e.m. Linear regression is shown with a 95% confidence interval. Source data

**Extended Data Fig. 7:**
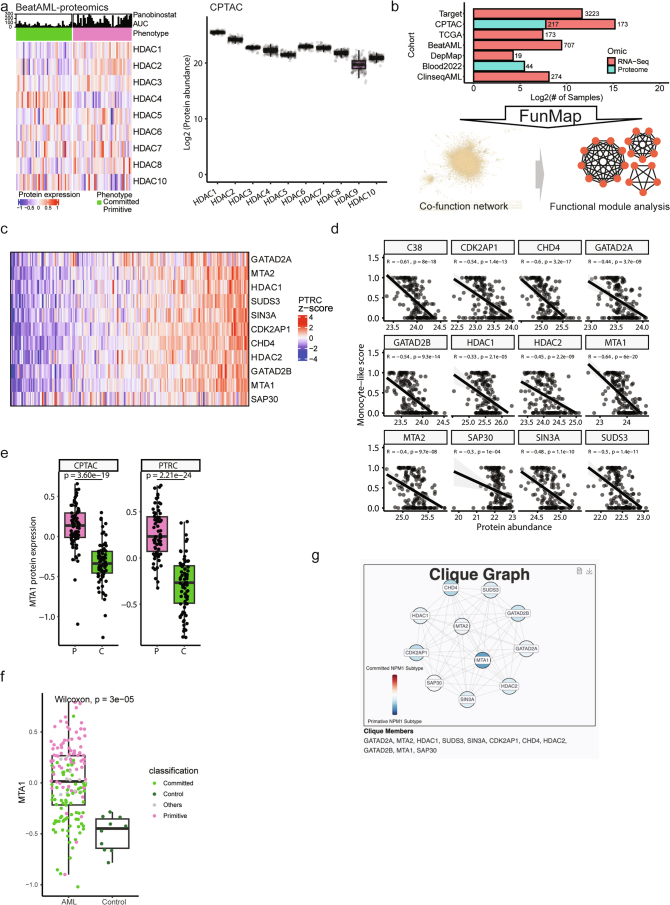
FunMap network analysis. **a**. Distribution of protein abundances for HDACs in the BeatAML-proteomics cohort. Protein abundances of HDAC proteins in the CPTAC AML cohort (n = 162 patients) are shown as a boxplot. **b**. Diagram depicting the generation of the AML-specific co-functional network from various datasets and functional modules using FunMap. **c**. Distribution of protein abundance of 11 genes within the C38 clique across the BeatAML-proteomics cohort. **d**. Spearman correlation between the protein abundance of 11 genes including MTA1 (red) within C38 and mono-like score in this cohort. Spearman rank correlation coefficients and unadjusted two-tailed p-values were computed. A linear regression line with a 95% confidence interval is also shown. **e**. Boxplots comparing protein expression of MTA1 between primitive and committed patients in both BeatAML-proteomics (n of primitive = 74 patients, n of committed = 74 patients) and CPTAC AML cohort (n of primitive = 75 patients, n of committed = 75 patients). Unadjusted p-values were derived from a two-tailed Student t-test. **f**. Distribution of protein abundances of MTA1 in the AML (n = 162 patients) and bone marrow control (n = 10 patients) samples from healthy donors. Unadjusted p-value is derived from a two-sided Wilcoxon rank-sum test. **g**. Screenshot of the FunMap website demonstrating the constituent and associated features of C38. Boxes represent the interquartile range (IQR) and whiskers extend to 1.5× the IQR or to the minimum and maximum values if no point exceeds that range. No outliers were removed from the plots. Source data

The dysregulation of HDACs may have therapeutic implications for the HDAC inhibitor panobinostat, which demonstrated notable efficacy in primitive AML in previous studies^16,33^. Panobinostat inhibits a wide range of class I, II and IV HDACs, although its primary targets in AML are insufficiently characterized. Candidates include HDAC1, HDAC2 and HDAC4, which exhibit the highest protein expression differences between primitive and committed AML (Fig. 6a and Extended Data Fig. 7a). HDAC1 and HDAC2 are putative targets because of their high abundance in AML (Extended Data Fig. 7a) and an inverse correlation with CD14^+^ monocytic differentiation (Fig. 6b). However, the expression of neither of these HDACs is strongly predictive of panobinostat response (area under the curve, AUC) in the BeatAML-proteomics cohort^16^ (Fig. 6a). Hypothesizing that panobinostat response depends on HDAC1/2 interactors, we applied FunMap^56^ to construct a cofunction network integrating our cohort with six external AML datasets (Extended Data Fig. 7b, Methods and Supplementary Table 30). Within this network, we applied the graph-theory-based iterative clique enumeration (ICE) algorithm^57^ and identified 274 fully connected cliques (Extended Data Fig. 7b and Supplementary Table 31).

We found seven cliques with elevated abundance in primitive AML and significant association with poor survival (Fig. 6c and Supplementary Table 32). Notably, C38 consists of HDAC1, HDAC2 and nine additional genes involved in complexes related to histone deacetylation, NuRD and Sin3. These 11 proteins were strongly positively correlated with each other in our cohort and the BeatAML-proteomics cohort (Fig. 6d and Extended Data Fig. 7c) but negatively correlated with panobinostat sensitivity (Fig. 6e). Among them, MTA1, a scaffold protein in the NuRD complex, showed the strongest correlation (Fig. 6f). In our cohort, MTA1 also demonstrated the most significant negative correlation with monocyte-like score (Extended Data Fig. 7d,e). At the protein level, MTA1 expression was significantly higher in AML as compared to healthy bone marrow controls (Extended Data Fig. 7f). We interrogated the role of *MTA1* in promoting panobinostat resistance through CRISPR–Cas9 knockouts (Supplementary Table 33) and in vitro studies. Deletion of *MTA1* restored sensitivity to panobinostat in both parental MOLM-14 and MONO-MAC-6 cell lines, demonstrating a strong dependency on MTA1 for survival (Fig. 6g,h). As hypothesized, overexpression of MTA1 in an MOLM-14 derived quizartinib-resistant cell line with known panobinostat sensitivity^16^ conferred resistance (Fig. 6i). We developed a website for interactive exploration of the AML FunMap (https://bzhanglab.github.io/funmap_aml/) (Extended Data Fig. 7g).

### Proteogenomic identification of therapeutic vulnerabilities in AML

**Fig. 7:**
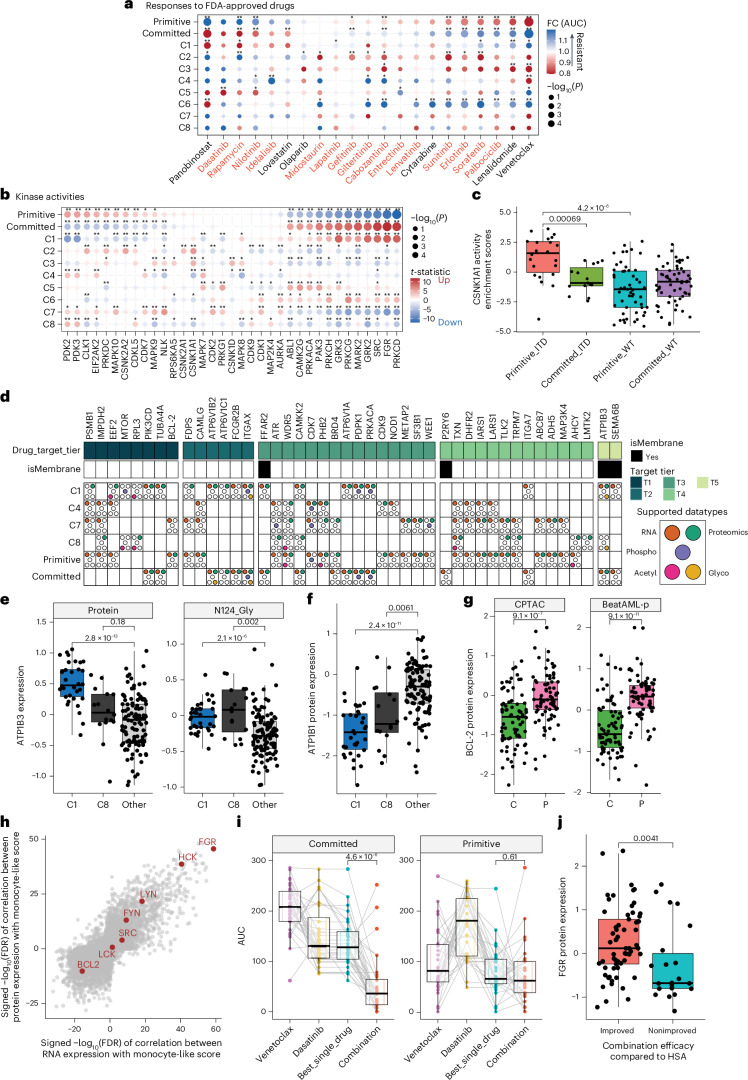
Proteogenomic identification of therapeutic vulnerabilities in AML. **a**, FDA-approved drugs showing significantly different sensitivity across predicted subtypes in the BeatAML-proteomics cohort. Kinase inhibitors are highlighted in red. **b**, Top five most significantly upregulated kinases for each subtype. **c**, Box plots comparing CSNK1A1 activity across four subgroups (*n* = 22, 15, 53 and 60 for primitive_ITD, committed_ITD, primitive_WT and committed_WT, respectively) defined by phenotype and *FLT3*-ITD mutation. *P* values were derived from a two-tailed Student *t*-test. **d**, Plots depicting top ten potentially druggable targets identified in C1, C4, C7 and C8 subtypes and primitive or committed phenotypes. **e**, Box plots depicting protein abundance of ATP1B3 and ATP1B3 N124 glycosylation in C1, C8 and other subtypes (*n* = 34, 16 and 112 for C1, C8 and other subtypes, respectively). *P* values were derived from a two-tailed Student *t*-test. **f**, ATP1B1 protein abundance in tumors classified in C1, C8 and other subtypes (*n* = 34, 16 and 112 for C1, C8 and other subtypes, respectively). *P* values were derived from a two-tailed Student *t*-test. **g**, Box plots comparing protein expression of BCL-2 between primitive and committed phenotypes in CPTAC (*n* = 75 for primitive and committed) and BeatAML-proteomics cohorts (*n* = 74 for primitive and committed). *P* values were derived from a two-tailed Student *t*-test. **h**, Plot depicting signed −log_10_ adjusted *P* value from Spearman correlation between the monocyte-like score with RNA or protein expression. SRC family kinases and BCL-2 are annotated in red. **i**, Box plots depicting AUC drug response values (lower values are associated with higher sensitivity from the BeatAML-proteomics cohort separated by primitive or committed phenotypes. AUCs for single agents venetoclax and dasatinib, as well as the best response of venetoclax and/or dasatinib, are shown (*n* = 32 and 31 for committed and primitive, respectively). *P* values were derived from a two-tailed Student *t*-test. **j**, Box plots compared the protein abundance of FGR in the BeatAML-proteomics cohort, where the venetoclax + dasatinib combination was more effective (lower AUC) than the best single agent (*n* = 281 and 106 for improved and nonimproved group, respectively). *P* values were derived from a two-tailed Student *t*-test. Boxes represent the IQR and whiskers extend to 1.5× the IQR or to the minimum and maximum values if no point exceeds that range. No outliers were removed from the plots. HSA, highest single agent. Source data

**Extended Data Fig. 8:**
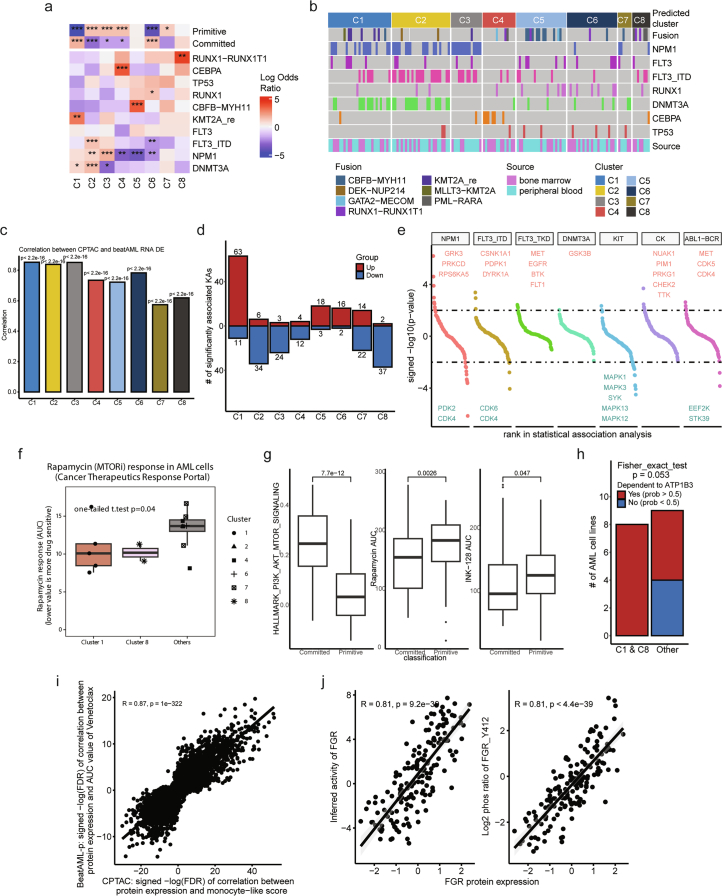
External projection of AML-8 subtypes. **a**. Enrichment of genomic aberrations across predicted subtypes in the BeatAML-proteomics cohort. (Asterisks indicate nominal p-value * = 0.05, ** = 0.01, *** = 0.001, two-sided Fisher exact test.). **b**. Oncoprint of key AML genomic alterations across predicted subtypes in the BeatAML-proteomic cohort. **c**. Pearson correlation of differentially expressed RNAs for each cluster identified in the CPTAC and the BeatAML-proteomics cohort. p-values are derived from Pearson correlation. **d**. Number of kinases with significantly dysregulated kinase activity (KA) in either up (activation) or down (repression) direction, across the AML-8 subtypes. **e**. Select kinases with differential activities across AML with driver genetic aberrations after adjusting for AML cellular hierarchy. Unadjusted p-values were derived from a two-tailed Student t-test. **f**. Response to rapamycin in DepMap AML cell lines with assignments to AML-8 subtypes. p-values were derived from a one-tailed Student t-test. The sample size (n) for each subtype is shown in the plot. The box plot’s centre line represents the median, with the lower and upper boundaries indicating the first and third quartiles, and whiskers extend 1.5 times the interquartile range from the lower and upper quantiles. **g**. (left panel) Activity of PI3K/MTOR signaling across BeatAML-proteomics samples stratified by primitive vs committed status (n = 130 patients). (middle and right panels) AUC of two MTOR inhibitors Rapamycin (n = 130 patients) and INK-128 (n = 131 patients) by committed / primitive status. Unadjusted p-values were derived from the two-sided Wilcoxon rank-sum test.The box plot’s centre line represents the median, with the lower and upper boundaries indicating the first and third quartiles, and whiskers extend 1.5 times the interquartile range from the lower and upper quantiles. **h**. Classification of AML cell lines based on their dependency on ATP1B3. Cell lines predicted to be C1 or C8 are compared to other AML-8 subtypes. Unadjusted p-values were derived from a two-tailed Fisher exact test. **i**. Plot depicting signed -log10 p-values derived from Spearman correlation between protein expression and monocyte-like signature in CPTAC AML (x-axis) and between protein expression and venetoclax AUC from the BeatAML-proteomics cohort. Spearman rank correlation coefficients and unadjusted two-tailed p-values were computed. **j**. Spearman correlation between protein abundance of FGR with inferred kinase activity of FGR (left panel) and FGR Y412 phosphosite abundance (right panel). Spearman rank correlation coefficients and two-tailed p-values were computed. A linear regression line with a 95% confidence interval is also shown. Source data

To investigate the therapeutic implications of protein-centric subtypes and AML cellular differentiation hierarchies, we leveraged drug response data from the BeatAML-proteomics cohort. We trained an RNA-based XGBoost classifier using 500 genes to predict subtype membership (Methods). Subtypes predicted in the BeatAML-proteomics cohort reflected mutation profiles and cellular hierarchy within the current study (Extended Data Fig. 8a,b), exhibited highly correlated RNA expression profiles (Extended Data Fig. 8c) and further revealed differential responses to FDA-approved drugs, particularly kinase inhibitors (Fig. 7a). We used PTM-SEA^58^ to identify further dysregulated kinase activities (Extended Data Fig. 8d). Kinase activity was found to associate with maturation (Fig. 7b) but some kinases were also influenced by genomic mutations when adjusted for cellular hierarchy (Extended Data Fig. 8e). For example, CSNK1A1 exhibited the compounding impact from *FLT3*-ITD and primitive subtype (Fig. 7c).

To systematically characterize therapeutic vulnerabilities, we applied our recently published approach^59^, which integrates tumor proteogenomics with cell line genetic perturbation (CRISPR) data to identify five tiers of drug targets on the basis of drug availability and development stage (Supplementary Table 34 and Methods). XGboost was used to map AML cell lines from DepMap^60^ to eight subtypes on the basis of RNA. Predicted drug targets were then nominated on the basis of protein overexpression in our cohort and CRISPR knockout dependency scores in cell lines (Supplementary Table 35–40 and Methods). Among all predicted potential targets, the top ten for subtypes C1, C4, C7 and C8 are summarized (Fig. 7d). Notably, mTOR is supported by phosphoproteome and acetylome data and by cell line dependency data in subtypes C1 and C8 (Fig. 7d). This was further validated through mTOR inhibitor rapamycin response in ex vivo individual-derived models and cell line data (Fig. 7a and Extended Data Fig. 8f), as well as sensitivity to rapamycin and another MTOR inhibitor in participant samples (Extended Data Fig. 8g). ATP1B3, a membrane protein, also emerged as a potential target for subtypes C1 and C8 (Fig. 7d). This nomination is supported by multiple omes (Fig. 7e), dependency data (Extended Data Fig. 8h) and the low expression of its synthetic lethal partner, ATP1B1 (Fig. 7f).

We also found BCL-2, the protein target of venetoclax, to be an overexpressed dependency in cell lines predicted to be primitive AML (Fig. 7d,g). A strong correlation was observed between venetoclax resistance and monocyte-like scores (Extended Data Fig. 8i), which corroborates the observed ex vivo drug response in the BeatAML-proteomics cohort (Fig. 7a). Next, we used correlation with monocyte-like AML scores as an indicator of genes putatively involved in venetoclax resistance. FGR exhibited the strongest positive correlation with monocytic differentiation (Fig. 7h) along with other Src family kinases such as HCK and LYN. Moreover, correlation analysis suggests that the FGR protein could serve as an indicator of FGR activity (Extended Data Fig. 8j). In the BeatAML cohort, combining dasatinib with venetoclax significantly improved responses in venetoclax-resistant committed AML (Fig. 7i). FGR was upregulated in models favoring the combination (Fig. 7j), suggesting it as a response biomarker for dasatinib and venetoclax.

## Discussion

AML is a complex disease at the genetic, phenotypic and clinical levels, which is reflected in its constantly evolving diagnostic, prognostic and therapeutic landscape^2,9^. Despite its size (*n* = 163 participants), our cohort captures diverse AML subtypes (*NPM1*-mutant, MDS-related, fusion-driven and *TP53*-mutant). Here, we sought to create a comprehensive multiomic atlas to illuminate the biology of primary samples from participants with AML. Specifically, we aimed to build upon existing genetic (for example, WHO^9^ and ELN^2^) and phenotypic (for example, Mito-AML^13^ and *NPM1* subsets^3^) classifications to provide a unified view of AML that uncovers novel pathobiology across multiple data modalities and may have translational utility to inform individual stratification and the deployment of targeted therapies.

We structured AML heterogeneity around two organizing principles: discrete protein-centric subtyping (AML-8) and continuous MYC or mTOR antagonism, validated by multiomic factorization (MOFA). AML-8 demonstrates that heterogeneity is driven by two primary axes—genotype (specifically *NPM1*, *CEBPA* and *RUNX1*–*RUNX1T1*) and cellular differentiation (distinguishing primitive from committed *NPM1*-mutant and MDS-related classes). While other classifications favor either genotype (Severens et al.) or differentiation (Jayavelu et al. and Pino et al.), AML-8 integrates both, although future reconciliation of these schemes is needed to determine their comparative clinical utility.

We also corroborated the identification of Mito-AML^13^, refining it to represent a subtype (C2 in our study) of primitive, *NPM1*-mutant and *FLT3*-ITD-mutant AML. Importantly, this revealed a novel subtype of *NPM1*-mutant AML (C3) characterized by outlier activity or expression of known oncogenes in AML *POU2F1*, *FOXC1* (ref. ^38^) and *HOXB8*. Comparing adult AML-8 to pediatric and normal cells remains a key future goal, despite data integration challenges. We identified MAP1A as a universal protein marker for primitive AML, extending from *NPM1*-mutant to MDS-related cases and correlating with venetoclax response. While prospective validation is required to establish predictive utility, MAP1A currently serves as a robust diagnostic marker for primitive, venetoclax-sensitive AML, further supported by its ability to distinguish leukemic stem cells from healthy counterparts.

We also characterized a series of PTMs (acetylation, phosphorylation, succinylation and glycosylation) in relation to the AML-8 and observed striking subtype-specific dysregulation of protein acetylation. Mitochondrial hyperacetylation characterizes the *CEBPA*-mutant enriched C4 subtype, whereas the C8 is marked by global hypoacetylation. The underlying mechanisms are also distinct. Mitochondrial acetylation is nonenzymatic^51^ and likely caused by increased acetyl-CoA levels, indicative of underlying metabolic changes. Conversely, hypoacetylation of C8 is associated with expression changes of HDACs and HATs. These findings reveal the critical interplay between PTMs and metabolism and identify acetylation dysregulation as a promising therapeutic target in AML.

Across most analyses, we observed a sharp antagonism between MYC and mTOR activity, reflecting the polarized relationship between primitive and committed AML. A sharp antagonism between MYC and mTOR mirrors the polarization of primitive versus committed AML, driving distinct metabolomic reprogramming. Primitive AML features upregulated OXPHOS and PS enrichment, whereas committed AML exhibits a glycolytic signature and PC enrichment. Conversely, GMP-like AML displays strong catabolic activity—driven by increased proteolysis and decreased eIF4 translation—and HexCer enrichment. These profiles reveal therapeutic opportunities targeting apoptosis, pyroptosis and ferroptosis in primitive AML, complementing existing MYC/mTOR strategies.

We used multiomic machine-learning tool ‘FunMap’ to map resistance mechanisms and therapeutic targets, validating findings in the external BeatAML cohort. Key insights include the identification of MTA1 as a driver of resistance to panobinostat and ATP1B3 as a novel target. We also demonstrated subtype-specific synergies, specifically showing that venetoclax plus dasatinib offers improved efficacy in committed AML because of elevated FGR and Src kinase expression.

This retrospective study has limitations impacting generalizability. The cohort, primarily of European descent with restricted healthy controls, does not capture full global diversity or all AML subtypes. International healthcare variability confounds survival data, while complex mutation patterns constrain statistical power, forcing trade-offs between clustering granularity and heterogeneity masking. Technical challenges included preanalytical variability (for example, sample purity and endogenous proteases), limitations in MS (for example, unquantified 2-HG) and the inherent inability of bulk profiling to distinguish regulatory changes from cell population shifts. Lastly, machine-learning subtype transfer is challenging and translational associations—such as MAP1A-associated venetoclax sensitivity—require mechanistic validation in independent cohorts.

## Methods

### Experimental model and study participant details

A total of 173 participants were included in this study. Institutional review boards at each tissue source site (TSS) reviewed protocols and consent documentation, adhering to Clinical Proteomic Tumor Analysis Consortium (CPTAC) guidelines and companies with all ethical regulations. Written consent was obtained from all participants.

Clinical data were obtained from TSS and aggregated by the CPTAC Biospecimen Core Resource (BCR; at the Pathology and Biorepository Core of Van Andel Research Institute). Data forms were stored as Microsoft Excel files. Participants with any prior history of other malignancies within 12 months or any systemic treatment (chemotherapy, radiotherapy or immune-related therapy) were excluded from this study (Supplementary Table 1).

### Normalization of PTMs

TMT-Integrator’s ratio reports (median-centered) on global proteome and PTM (phosphorylation, acetylation and glycosylation (site level)) datasets were used to build a simple linear regression model with all the samples’ global parent protein ratio as a predictor and their respective PTM ratio data as response. After the model is fitted, the residual values are taken as normalized PTM intensity.

### Removal of unwanted sources of variation and data normalization

RBCs are an unwanted source of contamination, especially in MS data acquisition of blood samples. During sample preparation, sample colors were recorded on the basis of their visual appearance as very red, red and light red. A darker color indicates more RBCs in the sample. To evaluate RBC contamination, we obtained 14 RBC marker genes from a previous study^61^ to derive an RBC contamination level score by summing up the protein expression intensities of the 14 marker genes and then scaling it to 0–1 across all samples (min–max normalization), where a higher RBC score suggests higher contamination. We further verified that RBC scores aligned well with sample colors. To eliminate the RBC contamination influence on protein data, limma’s removeBatchEffect function^62^ was used, taking RBC scores as a continuous batch effect and accounting for tumor purity and source sample type as covariates, that is, ~RBC + purity + sample_source. However, there is a known tendency of leukemia cells from different subtypes to be found in either the peripheral blood versus the bone marrow (peripheral tropism) and whether these resulting phenotypic differences represent a distinct biological state or an unwanted source of variation depends on the specific biological question being investigated. The effect of tumor purity on protein measurements is relatively modest; only 6% (583/9,808) are significantly (false discovery rate (FDR) < 0.10) associated with purity and tumor purity is not associated with cluster membership. The same adjustment was applied to RNA, PTM, metabolomics and lipidomics data. All downstream analyses use batch-corrected data or alternatively incorporate the same covariates directly into the statistical analysis. To facilitate reuse and enable different decisions regarding unwanted sources of variation, our data depositions include both uncorrected and batch-corrected data as part of the data release.

### WHO classification

A hierarchical method was developed to classify participants according to WHO criteria^9^. Participants with class-defining fusions (*PML*–*RARA*, *RUNX1*–*RUNX1T1*, *CBFB*–*MYH11*, *DEK*–*NUP214*, *ABL*–*BCR*, *KMT2A*–rearrangements and *NSD1*–*NUP98*) were first categorized. This is followed by classifications based on mutations (*NPM1* and *CEBPA*). Lastly, participants were identified as MDS if they had CK (MDS-cytogenetic) or had mutations in *ASXL1*, *SRSF2*, *SF3B1*, *U2AF1*, *ZRSR2*, *EZH2*, *BCOR* or *STAG2* (MDS-somatic). The remaining participants were categorized as AML with differentiation.

### EIF analysis

Translation initiation is known as the rate-limiting step in protein synthesis so we focused on the investigation of EIFs as representatives of protein synthesis^45^. A total of 42 EIFs (eliminating EIF pseudogenes) were identified in our proteome data.

Among all the EIFs, the EIF4F complex is regarded as the key regulator of the mRNA–ribosome recruitment phase of translation initiation. EIF4F deregulation has been shown to impact the translational efficiency of specific mRNAs, further affecting the synthesis of certain proteins^63^, such as oncoproteins in cancer. In this study, we built a linear regression model to identify the target proteins regulated by EIF4F in our AML cohort. The model is formulated as follows:

$$\mathrm{y\_prot\_i}\,=\,{\rm{\beta }}0+{\rm{\beta }}1* \mathrm{eif}\,+\,{\rm{\beta }}2* \mathrm{rna\_i}\,+\,{\rm{\beta }}3* \mathrm{eif}* \mathrm{rna\_i}\,+\,\in {\rm{i}}$$

where the response variable y_prot_i is the protein abundance of the query gene (target), eif is the protein abundance of the examined EIF, rna_i is the gene expression of target and eif*rna_i is the interaction term representing the joint effect of the examined EIF regulation and target gene expression on the target protein abundance. The underlying assumption of this model is that the protein abundance of a target is primarily regulated by the translation process, rather than a result of its gene expression.

The model was applied separately to EIF4F complex subunits EIF4A1, EIF4E and EIF4G1 to identify regulated targets. Targets were retained only if they met all three filters: positive EIF regulation effect (β1) on target protein expression, no significant effect of target gene expression (β2) on its protein and no significant combined influence of EIF regulation and target gene expression (β3) on target protein abundance. This yielded 40, 43 and 55 significant targets for EIF4A1, EIF4E and EIF4G1, respectively. Targets were pooled and an EIF4F regulation score was calculated as the median aggregated protein expression across all targets, with higher scores indicating greater protein synthesis activity.

### Deconvolution and immunological feature characterization

To estimate the cell type composition of each AML sample, a transcriptome-based deconvolution method previously described^26^ was used. In short, batch-corrected RNA count data were supplied to estimate cell type through a healthy bone marrow single-cell RNA-seq reference.

Raw count data were downloaded from NCBI’s Gene Expression Omnibus database with accession number GSE235063, focusing on malignant cells as identified in the original publication. The data were subsetted to include only primitive (HSCs), GMP and committed (CD16^+^ monocytes) from the diagnosis cohort to match the bulk data then downsampled to 500 cells per cell type to control for differences in cell type proportions. Deconvolution was then performed using BayesPrism^64^ with default parameters on the filtered count data. Moreover, LSC17, LinClass7, HLA II, cytolytic and inflammatory (iScore) scores were calculated on the basis of RNA-seq data following previous publications^4,32,65,66^.

To estimate primitive and committed scores, a signature was derived from a previous study^3^. Significantly upregulated and downregulated genes were used as inputs for singscore^67^ to calculate a score corresponding to primitive and committed phenotypes. Participants were categorized as primitive or committed if they had scores higher than the median of all samples.

### DE and abundance analyses

R Bioconductor package limma^62^ was used to fit a linear regression model between sample groups for data in the log_2_ scale. After model fitting, the regression coefficient is the log_2_FC between comparison groups (mean difference between two groups) and the *P* value and adjusted *P* value associated with the moderated *t* statistic (p.mod and q.mod) calculated with the eBayes function are the resultant significance measurement. Voom was used to normalize RNA count data for DE analysis, with other steps performed similarly.

The effect of mutations on proteome and PTMs was assessed by *t*-test comparing the expression between mutant and wild-type samples. For *NPM1* mutations, interactors from BioGRID were used to filter cross-analysis of DE at RNA and protein levels. Subcellular locations were obtained from The Human Protein Atlas ‘main location’ and simplified into three groups: cytoplasm main, nucleus main and others. Proteins with a single listed compartment were assigned directly; those with multiple compartments were classified by dominant location, with equal cytoplasm and nucleus counts classified as ‘others’ and missing annotations labeled ‘NA’.

### Pathway and signature enrichment analyses

For functional characterization of subtyping results, we used single-sample gene set enrichment analysis (ssGSEA) and fast gene set enrichment analysis^68^ to calculate the normalized enrichment scores of cancer-relevant gene sets from Hallmark, C2 and C5 MsigDB pathways. ssGSEA was applied to both transcriptomics and proteomics using the same parameters, enabling the quantification of pathway activity at the individual sample level. Gene sets with an FDR < 0.05 were considered significantly enriched.

### MOFA

We performed three sets of MOFAs^69^. Three MOFAs were performed using default hyperparameters. First, the top 50 most variable RNA and protein features (batch-corrected) were used to infer 30 factors. Second, METABO-MOFA incorporated metabolomics (combined RP and HILIC, matched samples only) and lipidomics (combined positive and negative modes). Third, PTM-MOFA combined RNA, global proteome, phosphorylation and acetylation; phosphorylation and acetylation data were normalized against parent proteins and batch-corrected, with acetylation data non-median-centered. Factors were interpreted using standard MOFA2 R functions (plot_variance_explained, correlate_factors_with_covariates and plot_weights). Additionally, the overrepresentation test was performed using clusterProfiler^70^ with the Gene Ontology (GO) cellular component gene set obtained from MSigDB^71^.

### TF activity inference

TF activities were inferred from bulk RNA-seq data using the packages SCENIC^36^ and decoupleR^72^.

For decoupleR, we used curated TF gene regulon libraries (DoRothEA^73^ and CollecTRI^74^) for inferring regulon activity. Standard processing and default settings were used to fit a univariate linear model using the function decoupleR::run_ulm. This method estimates a TF score for each participant and a Wilcoxon test was performed to determine statistical significance of TF activity between participant subtypes.

### SNF result clustering

The SNF method^75^ was applied using the R package SNFtool. SNF clustering used participants with both imputed RNA and protein expression data. To identify robust clusters, RNA and protein features were independently ranked by s.d., with input features varied from 10% to 100% of the most variable features and cluster numbers evaluated from 3 to 10. All SNF analyses used consistent parameters: Euclidean distance, α = 0.5, 20 neighbors, ten diffusion iterations and spectral clustering using SNFtool.

### Construction of FunMap network

The FunMap package was obtained from GitHub (https://www.github.com/bzhanglab/funmap) and used to construct the AML-specific FunMap network. The public AML RNA-seq and proteomic datasets (Supplementary Information) were used as input and the log-likelihood ratio (LLR) was calculated for each gene pair, with functional associations determined using an LLR threshold of 3.912, which represents a 50-fold likelihood ratio. This resulted in an AML FunMap network comprising 13,729 genes and 134,100 edges (Supplementary Table 30). The integration of proteomics data was a major contributor to the high functional relevance of AML FunMap.

Fully connected cliques were extracted from the network using a graph-theory-based ICE algorithm^57^ (minimum size = 5, average abundance).

### Visualization of FunMap network

We created a website for users to visualize and explore the AML FunMap network through cliques and gene neighborhoods. A clique represents a densely interconnected gene set, while a gene neighborhood constitutes the top 50 nearest neighbors to a gene of interest determined by a random walk. After selection of a subnetwork, the website visualizes the connections between the different genes in an interactive graph. The website also creates tables of the subnetwork’s association with features relevant to AML, including GO terms, *NPM1* subtype and overall survival. Users can interact with the table to color the node graph by associating each node to the selected feature (Extended Data Fig. 6g). The AML FunMap website is publicly available from GitHub (https://bzhanglab.github.io/funmap_aml/).

### Survival analyses

Survival analysis was performed using the R package survival^76^. The ‘survfit’ function was used to calculate the Kaplan–Meier curve and calculate the nonparametric statistical differences between subtypes. The R package survival was then used to plot the survival curve.

### Lipidomics differential correlation analysis

Pairwise Spearman correlations were computed for all lipid species within each phenotype (committed, primitive or GMP). The difference in correlations for each phenotype versus the other two phenotypes was computed and the top 200 most differentially and positively correlated lipid–lipid pairs for each phenotype were plotted. The percentage of differential correlations from lipid class relationships within phenotypes was calculated and plotted if a given phenotype had class–class relationships not present in the other two phenotypes.

### ICA

ICA was used to factorize metabolomics data into statistically independent components. First, data were filtered to remove metabolites with few data points and *k*-nearest neighbor approach was used for imputation. The data were log-transformed and *z* score was normalized to minimize skewness and ensure comparability across metabolites. ICA was performed using the FastICA algorithm^77^ and models were assessed using the Bayesian information criterion (BIC) to assess the best number of factors to use.

$$\mathrm{BIC}=k\mathrm{ln}(n)-2\mathrm{ln}(L(\hat{\theta }))$$

where *k* is the number of parameters estimated by the model, *n* is the sample size and $L(\hat{\theta }$) describes the maximized value of the likelihood function for the model. A lower BIC value indicates a better model with balanced accuracy and simplicity.

### Inference of clusters in BeatAML, BeatAML-proteomics and DepMap

XGBoost method was applied to RNA expression data (counts per million matrix) to train an RNA-based classifier. RNA-based prediction was chosen over protein-based prediction because RNA-seq data are more widely available across prior studies, enabling integration of external datasets including AML cell line data. RNA-based classification yielded predicted clusters in BeatAML with higher similarity to the original AML-8 than protein-based clusters (significant increases Δ*R* > 0.2 for C3, C7 and C8). RNA-based and protein-based predictions produced clusters with highly similar phenotypes (*R* = 0.79–0.98), except C3, C7 and C8, which were poorly predicted by the protein classifier. Feature selection used the minimum redundancy maximum relevance algorithm; a 500-gene feature group was selected through fivefold cross-validation based on highest area under the receiver operating characteristic curve (AUROC), achieving median AUROC > 0.9 across all subtypes. This model classified samples from the BeatAML-proteomics cohort (210 participants with AML with quantitative proteome, phosphoproteome, whole-exome sequencing (WES), RNA-seq and ex vivo drug sensitivity data for >100 small-molecule inhibitors) into the eight predefined subtypes.

### Kinase activity inference analyses

We collected known kinase–substrate relationships (KSRs) from the PhosphoSitePlus database and the training data of GPS 5.0. Additionally, predicted relationships with scores > 5.0 were downloaded from NetworKIN^78^. We remapped the positions of these substrates on the basis of phosphorylated peptides and gene symbols to align with our reference database. In cases where a phosphorylated peptide mapped to multiple protein isoforms derived from the same gene, all mapped isoforms were designated as the substrates of the specific kinase. In total, 118,749 KSRs were collected between 424 kinases and 58,571 phosphorylation sites. Subsequently, PTM-SEA^58^ was used to infer the kinase activity with the unnormalized phosphoproteomic data as input. Phosphorylation sites with missing values in more than 50% of samples were filtered out and the following key parameters were used: weight = 0, statistic = ‘area.under.RES’, output.score.type = ‘NES’, nperm = 1000 and min.overlap = 5.

### Predicting effective drug targets

Details of our method for prioritizing overexpressed cancer dependencies as effective drug targets have been previously reported^59^. CRISPR gene effect scores from Broad Achilles and Sanger SCORE combined screens (CRISPR_gene_effect.csv) and drug response data from the Cancer Therapeutics Response Portal (CTRP version 2.0) were downloaded from DepMap for AML cell lines. RNA-seq-based subtype classifiers were applied to AML cell lines; subtypes C1 and C6 defined the committed phenotype while subtypes C2, C3 and C7 defined the primitive phenotype. Subtypes C1, C4, C7 and C8 had sufficient cell lines with CRISPR data for loss-of-fitness analysis. A one-sample, one-tailed *t*-test identified omics candidates (RNA, protein, activating phosphosite, kinase activity, acetyl site, ubiquitin site and glycosite) associated with significantly reduced growth upon gene knockout. Candidates were defined as therapeutic targets if they were significantly increased in tumors for a given subtype or differentiation state (adjusted *P* ≤ 0.05, Wilcoxon rank-sum with Benjamini–Hochberg correction) and had a CRISPR gene effect score significantly below zero (*P* ≤ 0.05).

### In silico validation of cluster prediction

This was tested in silico by comparing response in both ex vivo individual-derived models from the BeatAML-proteomics cohort and AML cell lines to an approved MTOR inhibitor, rapamycin, from the Cancer Therapeutics Response Portal. Models and AML cells assigned to subtypes C1 and C8 were more sensitive to mTOR inhibition than others.

### Panobinostat structural docking analysis

The PDB file of human HDAC1 was downloaded from the AlphaFold database (https://alphafold.ebi.ac.uk/) and the Mol2 file of panobinostat was downloaded from the ZINC database (https://zinc.docking.org/). AutoDock4 was applied for the docking simulation between HDAC1 and panobinostat. The protein structure was cleaned by removing heteroatoms and water molecules, followed by the addition of polar hydrogens using AutoDockTools^79^. The whole protein was taken as the search space to include all potential interactions and the conformation with the lowest binding energy was selected. PyMOL 2.5.0 was used for the visualization.

### CRISPR–Cas9 *MTA1* inactivation by individual sgRNAs

Inactivation of *MTA1* was carried out by cloning sgRNAs into plentiCRISPRv2 (Addgene, 52961) plasmid. This plasmid contains two expression cassettes, hSpCas9 and the chimeric gRNA. gRNA sequences (Supplementary Table 33) were obtained from Synthego and oligos with 5′ overhang for cloning into lentiCRISPRv2 were manufactured by Thermo Fisher Scientific. The BsmBI-digested, dephosphorylated vector was gel-purified and ligated with annealed, phosphorylated oligonucleotides. Plasmid was amplified in Stbl3 bacteria, purified and lentivirus-generated in HEK 293T/17 cells. Cells (1 × 10^6^) were transduced with 0.5–1 ml of viral supernatant in 2 ml of growth medium with 10 mM HEPES and 8 µg ml^−1^ polybrene in six-well format and centrifuged at 2,600 rpm for 2 h at 35 °C. MOLM-14 (courtesy of Y. Matsuo’s lab, University of Tokyo) and MONO-MAC-6 (courtesy of J. Maxson’s lab, Oregon Health and Science University) cells were selected with 2 µg ml^−1^ puromycin for 5–7 days and cultured 14 days before panobinostat sensitivity testing. *MTA1* downregulation was confirmed by western blot (anti-MTA1; D17G10, 5646, Cell Signaling; 1:1,000).

### Generating *MTA1*-overexpressed cell lines

HEK 293T/17 cells were transfected with pTFORF2895 (Addgene, 144355) to generate MTA1 lentivirus as described above. Transduced quizartinib-resistant MOLM-14 cells^80^ were selected in puromycin for 5–7 days and cultured for an additional 10 days before assessing overexpression of *MTA1* by western blotting and panobinostat sensitivity. Two *MTA1*-overexpressed cell lines were generated.

### Panobinostat inhibitor studies

Panobinostat was purchased from Selleck Chemicals, reconstituted in DMSO and stored at −80 °C. *MTA1*-knockout or *MTA1*-overexpressed cells were seeded into 384-well assay plates using a Multidrop TM Combi reagent dispenser (Thermo Fisher Scientific) at a density of 1,000 cells per well in 50 μl of RPMI medium supplemented with 10% FBS, 2% L-glutamine, 1% penicillin–streptomycin and 0.1% amphotericin B. Panobinostat was dispensed into the plates with an HP D300e Digital Dispenser (Tecan) at increasing concentrations. The final concentration of DMSO was ≤0.1% in all wells. All conditions were plated with six replicates. After 3 days of culture at 37 °C in 5% CO_2_, cell viability was measured using a methanethiosulfonate (MTS)-based assay (CellTiter96 Aqueous One Solution, Promega) and absorbance (490 nm) was read at 4 h after adding MTS reagent using a BioTek Synergy 2 plate reader (BioTek). MTS absorbances of inhibitor-treated wells were normalized to those of untreated cells as previously described^80^. GraphPad Prism 8 was used to model dose-specific, normalized cell viability values with four-parameter logistic regression curves to determine half-maximal inhibitory concentrations.

### Genomic and transcriptomic sample acquisition and processing

Biospecimen kits were manufactured and distributed to tissue source sites by the CPTAC BCR at the Van Andel Research Institute. AML samples required 5.0 × 10^6^–10.0 × 10^6^ cells per vial with >20% blasts in bone marrow aspirate or >50% in peripheral blood. Matched buccal swabs served as germline controls. Cell pellets were shipped by cryoport (<−140 °C) to the BCR, inspected and stored in liquid nitrogen. Pathologists verified blast percentage; disease-specific experts classified samples per FAB criteria. Matched pellets were used for DNA and RNA isolation (stored at −80 °C) and proteomics. Nucleic acids were shipped on dry ice and pellets by cryoport to respective characterization centers.

### Specimen processing standard operating procedures

Primary AML samples (bone marrow aspirate or peripheral blood) were collected in acid citrate dextrose tubes; the interval from collection to freezing did not exceed 8 h. Mononuclear cells were isolated by density gradient centrifugation and washed with PBS. Viable cell counts were performed and a quality control (QC) cytospin/smear slide was prepared for pathological review. Aliquots of 5–10 million cells were pelleted, frozen overnight at −80 °C and then transferred to liquid nitrogen vapor phase for long-term storage. Three buccal swabs were collected per donor for germline DNA.

### Genomic DNA and total RNA extraction

DNA and RNA were coisolated from tumor specimens using Qiagen QIAsymphony kits. Germline DNA was extracted from buccal swabs. DNA was quantified by Qubit dsDNA BR assay; RNA quality was assessed by NanoDrop 8000 and Agilent Bioanalyzer (RNA integrity number (RIN) ≥ 7 required for RNA-seq). Sample identity was confirmed using the Illumina Infinium QC array (15,949 markers).

### PCR-free whole-genome sequencing (WGS) library preparation

Genomic DNA (350 ng) was sheared by Covaris ultrasonication to ~385 bp and size-selected by solid-phase reversible immobilization (SPRI) cleanup. Libraries were prepared using KAPA Hyper Prep (without amplification) with unique dual-indexed forked adaptors (Integrated DNA Technologies), quantified by qPCR on an Agilent Bravo, normalized to 1.7 nM and pooled into 24-plexes.

### PCR-free WGS cluster amplification and sequencing

Pooled libraries were cluster-amplified on Illumina cBot and sequenced on HiSeq X (≥15× coverage, 151-bp paired-end reads). Demultiplexing and alignment were performed by the Picard pipeline.

### WGS PCR plus library preparation

For samples with lower DNA yields, 100 ng genomic DNA was sheared to ~385 bp, prepared with KAPA Hyper Prep with ten cycles of PCR amplification using Roche adaptors, quantified by qPCR, normalized to 2.2 nM and pooled into 24-plexes.

### WES library construction

WES libraries were constructed from 20–250 ng of genomic DNA following Fisher et al. with modifications. Briefly, palindromic forked adaptors with unique dual-index barcodes (Integrated DNA Technologies) replaced standard Illumina adaptors and Kapa Hyper Prep reagents were used throughout. Postenrichment SPRI elution was reduced to 30 µl with added vortexing. Up to 96 libraries were pooled and hybridized using Illumina’s Nextera exome kit on an Agilent Bravo liquid handling system, with minor protocol modifications for automation. Postcapture libraries were quantified by qPCR on Agilent Bravo and normalized to 2 nM. Libraries were sequenced on HiSeq 4000 (paired 76-cycle runs, two eight-cycle index reads) to ≥150× on-target coverage. Raw data were demultiplexed, trimmed and aligned to GRCh38/hg38; validated BAMs were used for variant calling.

### Total RNA-seq library construction

RNA integrity and concentration were assessed on an Agilent TapeStation (RIN > 8.0 required for fresh-frozen; DV200 and fragment size determined for FFPE). Target input was >500 ng µl^−1^. Indexed libraries were sequenced on Illumina HiSeq 4000 (paired-end 100 bp, ≥150 million reads per sample, >90% mapped target). Quality was assessed by mapping to hg38, evaluating coding region coverage, rRNA content, gene expression breadth and housekeeping gene expression. Samples were bioinformatically clustered against reference tumor types; atypical samples were single-nucleotide polymorphism (SNP)-typed for confirmation. FASTQ files were uploaded to the Genomic Data Commons (GDC) repository.

### miRNA-seq library construction and sequencing

miRNA-seq libraries were prepared and sequenced identically to total RNA-seq, with the same QC pipeline including clustering, expert review and SNP typing of atypical samples.

### Illumina Infinium MethylationEPIC BeadChip array

DNA methylation was profiled using the Illumina MethylationEPIC array (>850,000 CpG sites) from 250 ng of bisulfite-converted DNA. Data were processed through an automated genotype calling pipeline.

### Sample processing for protein extraction and tandem LysC/trypsin digestion

Cell pellets (5–10 million cells) were lysed in an 8 M urea buffer with protease and phosphatase inhibitors. Proteins were clarified by centrifugation, quantified by BCA assay, reduced with DTT, alkylated with iodoacetamide, diluted and sequentially digested with LysC (1:50, 2 h) and trypsin (1:50, 14 h). Peptides were acidified, desalted on a C18 solid-phase extraction column and dried.

### TMT labeling of peptides

Desalted peptides (250 µg per sample) were labeled with TMT 18-plex reagents (500 µg) in HEPES buffer, quenched with hydroxylamine and pooled across 13 plexes including a common reference channel. Labeled peptides were desalted on C18 and dried. TMT-labeled peptides (~4.5 mg per set) were fractionated by bRPLC on an Agilent Zorbax Extend-C18 column (4.6 × 250 mm, 3.5 µm) using ammonium formate (pH 10)–acetonitrile gradients, concatenated into 24 fractions. A 5% aliquot of each fraction was used for global proteomics; the remainder was reserved for phosphopeptide enrichment.

### Enrichment of phosphopeptides by Fe immobilized metal ion affinity chromatography (IMAC)

The remaining 95% was concatenated into 12 fractions and subjected to Fe^3+^-IMAC phosphopeptide enrichment using Fe^3+^-conditioned Ni-NTA beads. Phosphopeptides were eluted with potassium phosphate buffer, desalted on C18 stage tips and dried.

### Immunoaffinity purification of acetylated peptides

IMAC flowthrough fractions were concatenated into four fractions, reconstituted in immunoaffinity purification (IAP) buffer and incubated with anti-acetyllysine antibody beads (Cell Signaling PTMScan). Acetylated peptides were eluted with 0.15% TFA, desalted on C18 stage tips and dried.

### Enrichment of intact glycopeptides by MAX columns

IAP flowthrough was desalted on C18 columns, reconstituted in 95% acetonitrile and 1% TFA and enriched for intact glycopeptides using OASIS MAX solid-phase extraction. Glycopeptides were eluted with 50% acetonitrile and 0.1% TFA, dried and reconstituted for LC–MS/MS.

### LC–MS/MS for global proteome, phosphoproteome, acetylproteome and glycoproteome

Global, phosphorylated and acetylated proteome fractions were separated on in-house packed C18 columns (25 cm, 1.9 µm; ReproSil-Pur) at 200 nl min^−1^ and analyzed on an Orbitrap Eclipse with FAIMS (compensation voltages: −45, −60 and −75). Data-dependent acquisition used higher-energy collision-induced dissociation (HCD) fragmentation (32% collision energy (CE)) with 120,000 MS1 and 50,000 MS2 resolution. Glycopeptides were analyzed on an Orbitrap Fusion Lumos with Easy nLC 1200 using HCD (35% CE) with 60,000 MS1 and 50,000 MS2 resolution and a 2-s cycle time.

### Metabolite extraction and MS analysis

Metabolites were extracted from 5 × 10^6^–20 × 10^6^ cells by modified Folch extraction (chloroform, methanol and water, 8:4:3). The hydrophilic layer was dried and resuspended in 4:1 methanol and water. Samples were analyzed by RP LC and HILIC LC–MS/MS in positive mode on a Waters Acquity ultrahigh-performance LC (UPLC) coupled to a Thermo Q Exactive Plus. Solvent blanks, standards and pooled controls monitored quality throughout.

### Complex lipid extraction and MS analysis

The organic layer from Folch extraction was dried, reconstituted in 9:1 methanol and chloroform and analyzed on a Waters Acquity UPLC/Thermo Lumos Orbitrap using a CSH C18 column in both positive and negative ionization modes. Full MS scans (120,000 resolution, 200–1,800 *m*/*z*) with data-dependent HCD fragmentation were acquired.

### Primary genomics data processing and quality control

WGS, WES and RNA-seq data were processed using the TPO pipeline (https://github.com/mctp/tpo). DNA reads were trimmed with BBMap bbduk and aligned to GRCh38 with BWA-mem, before undergoing GATK best-practices QC (indel realignment, base quality score recalibration and duplicate removal by Sentieon). Tumor–normal genotype concordance was verified by DNAscope. Methylation β values were obtained using the SeSAMe-based harmonization workflow from GDC.

### Somatic mutation calling

Somatic variants were called from WES using TNscope with default settings and custom postfilters on the basis of allele frequency, coverage, tumor and normal limits of detection, strand bias, problematic regions and homopolymer repeats. Stricter filters were applied for tumor-only samples. All variants were manually reviewed.

### RNA quantification and gene fusion calling

RNA-seq reads were aligned with STAR (GRCh38, Gencode 42/Ensembl 108 splice junctions) using crisp_align and quantified with Kallisto through crisp_quant, before detecting gene fusions with crisp_codac. All tools were run with default settings within the TPO pipeline.

### SV calling

SVs were called from WGS using MANTA and TNscope within TPO (cords-structural), with record-level and sample-level quality filters. Calls were stratified by confidence level on the basis of split-read and spanning read-pair support. *FLT3*-ITD was detected by Pindel applied to WGS and WES reads aligned to GRCh38. Calls with <5 supporting reads were removed; remaining calls were manually reviewed in Integrative Genomics Viewer.

### Copy-number variation analysis

Copy-number variations were analyzed from WGS using cords_cnvex within TPO. The log-ratio coverage and B-allele frequencies were GC-corrected, jointly segmented by circular binary segmentation and subjected to purity and ploidy inference with manual review of candidate solutions.

### Protein quantification

Protein quantification used TMT-Integrator with ratio-based integration. Peptide spectral match-level ratios to a common reference were computed from MS2 reporter ions, aggregated to peptide and protein levels, converted to an intensity scale and median-centered across samples.

### Quantification of intact glycopeptides and glycosite localization

Glycopeptides were identified using MSFragger-Glyco (version 3.8) in FragPipe using a 252-composition human *N*-glycan database restricted to N-X-S/T. Diagnostic oxonium and *y* ions were used for spectral validation. Glycan assignment and FDR filtering (1%) were performed in PTM-Shepherd. TMT-Integrator generated quantification reports at gene, protein, peptide, site and multimass levels.

### Customized searches of proteomics data

Semitryptic peptide ratios were estimated from label-free QC data searched with FragPipe (LFQ-MBR, semienzymatic). Succinylation was assessed in IMAC phosphoproteome data using MSFragger-Labile in FragPipe, with PeptideProphet rescoring, PTMProphet localization (probability ≥ 0.75) and TMT-Integrator quantification.

### Public RNA-seq and proteomic datasets for FunMap analyses

External datasets included BeatAML (707 samples), TCGA-AML (173 samples, cBioPortal), TARGET-AML (2,762 AML, 442 normal, 18 cell lines, GDC), Clinseq-AML (274 participants, Wang et al.), Blood2022 proteomics (44 participants, Kramer et al.) and DepMap 22Q2 cell line RNA-seq. TCGA, TARGET-AML and DepMap matrices were upper-quantile-normalized and log_2_-transformed before FunMap model training.

### Statistics and reproducibility

Our study cohort contained 173 treatment-naive AML samples from 103 female and 70 male participants with a median age of 60 years (range: 18–87), collected from Russia (81), Bulgaria (37), U.S. (19), Poland (7), Romania (5) and other countries (24). No statistical method was used to predetermine sample size. We summarized preliminary participant characteristics in (Supplementary Table 1). Tumor samples were collected from bone marrow as the preferred method and peripheral blood when needed, while matched normal samples were from buccal swabs on the cheek. For participants whose samples were collected from both sources, we selected the protein sample that matched the source of transcriptomics (Extended Data Fig. 1a,b). Source sample type (that is, bone marrow versus peripheral blood) was treated as an unwanted source of variation (batch effect) and removed before downstream analysis. The AML molecular landscape was explored at multiple omic layers, including WGS (173 tumors, 140 normals), WES (173 tumors, 161 normals), RNA-seq (172 tumors), proteomics including PTMs (162 tumors), metabolomics (91 tumors) and lipidomics (96 tumors) (Supplementary Table 1). Data collection and analysis were not performed blind to the conditions of the experiments but randomization was incorporated for different omic layers. All manuscript’s analyses were based on the genetic sex inferred from sequencing data, cross-referenced with the reported sex in the clinical records. Because of sample size, no sex-specific results are reported. Details of genomics, transcriptomics, proteomics, metabolomics and lipidomics sample acquisition and processing, as well as genomics, transcriptomics, proteomics and metabolomics data processing, are described in the Supplementary Information. Nonparametric statistical tests were performed if possible; otherwise, data distribution was assumed to be normal but this was not formally tested. No data were excluded. The analysis presented was built on samples with complete data across multiple omic layers (Extended Data Fig. 1b).

### Reporting summary

Further information on research design is available in the Nature Portfolio Reporting Summary linked to this article.

## Supplementary information

Supplementary Information List of consortium members.

Reporting Summary

Supplementary Tables 1–40 Supplementary Tables 1–40.

## Source data

Source Data Fig. 1 Statistical source data.

Source Data Fig. 2 Statistical source data.

Source Data Fig. 3 Statistical source data.

Source Data Fig. 4 Statistical source data.

Source Data Fig. 5 Statistical source data.

Source Data Fig. 6 Statistical source data.

Source Data Fig. 6 Unprocessed western blots and/or gels.

Source Data Fig. 7 Statistical source data.

Source Data Extended Data Fig. 1 Statistical source data.

Source Data Extended Data Fig. 2 Statistical source data.

Source Data Extended Data Fig. 3 Statistical source data.

Source Data Extended Data Fig. 4 Statistical source data.

Source Data Extended Data Fig. 5 Statistical source data.

Source Data Extended Data Fig. 6 Statistical source data.

Source Data Extended Data Fig. 7 Statistical source data.

Source Data Extended Data Fig. 8 Statistical source data.

## Data Availability

Harmonized genomic, transcriptomic, proteomics (raw MS files and processed data files of global proteomics and PTMs), metabolomics, lipidomics, methylation and clinical data files generated for this AML cohort can be accessed through GDC (https://portal.gdc.cancer.gov; project identifier: CPTAC-3). The raw proteomic data and the processed and harmonized proteogenomic data for the study cohort are available through the Proteomic Data Commons under accession numbers PDC000554, PDC000555, PDC000556, PDC000557, PDC000558, PDC000559, PDC000560, PDC000561 and PDC000562. The dataset generated and analyzed during this study is available through the Database of Genotypes and Phenotypes (dbGaP) under accession number phs001287.v22.p7, with controlled access to protect participant privacy and in accordance with the informed consent under which the data were collected. Access requests can be submitted through the dbGaP Authorized Access portal (https://dbgap.ncbi.nlm.nih.gov), where they are reviewed by the NCI data access committee (NCIDAC@mail.nih.gov), with approval typically granted within 4–6 weeks to qualified researchers who agree to the Data Use Certification terms. Histopathology images can be accessed through The Cancer Imaging Archive (10.7937/TCIA.2019.B6FOE619) and the Imaging Data Commons (https://portal.imaging.datacommons.cancer.gov/explore/filters/?collection_id=CPTAC&collection_id=cptac_aml). Additional processed data are provided in the Supplementary Information. Source data are provided with this paper.

## Code availability

This paper does not report original primary software tools. Analysis scripts and notebooks used for this study are open-source and available from GitHub (https://github.com/Nesvilab/CPTAC_AML). Any additional information required to reanalyze the data reported in this work paper is described in the Methods and available upon request from the corresponding authors. Functional network results for the AML cohort are available from GitHub (https://bzhanglab.github.io/funmap_aml).
