# Supplementary material for: Integrated proteogenomic and metabolomic profiling of acute myeloid leukemias to identify molecular subtypes and associated therapy targets
Source: Nat Cancer. 2026 Jun 12;7(6):993–1015. doi: 10.1038/s43018-026-01175-6 (PMC13309276; doi:10.1038/s43018-026-01175-6)
Supplement: Supplementary file 1 — List of consortium members. [file 43018_2026_1175_MOESM1_ESM.pdf]

nature cancer

Supplementary information https://doi.org/10.1038/s43018-026-01175-6

Integrated proteogenomic and metabolomic profiling of acute myeloid leukemias to identify molecular subtypes and associated therapy targets

In the format provided by the authors and unedited

Clinical Proteomic Tumor Analysis Consortium Members

Haitham Abdelhakim[20], Shelby Abts[20], Anupriya Agarwal[21], Eunkyung An[16], Veera Baladandayuthapani[31], Anand Basu[19], Garana Belinda[5], William Bocik[32], Melissa Borucki[33], Shuang Cai[19], Stancioaica Maria Camelia[34], Steven Carr[35], Patricia Castro[9], Daniel Chan[13], Jin Chen[1,12], Arul M. Chinnaiyan[1,12,27,28], Hanbyul Cho[2,12], Shih-Chun A. Chu[1,2,12], Rosalie K. Chu[5], Marcin P. Cieslik[1,2,12,^], Chaevien S. Clendinen[5], Simona Colantonio[33], Reese Crispen[33], Diwaker Davar[36], Yamei Deng[2], Saravana M. Dhanasekaran[1,12], Rajiv Dhir[36], Li Ding[24,25], Marcin Domagalski[19], Yongchao Dou[3,4], Jennifer Dunlap[21,22], Josie G. Eder[5], Nathan J. Edwards[18], John M. Elizarraras[8,9], John Evangelista[37], Brenda Fevrier-Sullivan[33], Javier E. Flores[5], Rafael Fonseca[38], John Freymann[33], Victoria Fulidou[39], Sharon Gaheen[33], Pencho Georgiev[34], Gad Getz[35], Lidia Gil[40], Michael A. Gillette[35], Andrew K. Godwin[20], Charles A. Goldthwaite Jr., Vladislav Golubkov[39], Mahnoor N. Gondal[2,12], Sara J.C. Gosline[5,30], Ramaswamy Govindan[14], Anthony Green[36], Marina A. Gritsenko[6], Leanne E. Henry[1,12], Michael Holck[19], Noshad Hosseini[2,12], Galen Hostetter[17], Yi Hsiao[2], Joel Hsu[38], Lan Huang[41], Omar M. Ibrahim[14,15], Michael Ittmann[9], Eric Jaehnig[9], Raghav Jain[5], Xiaojun Jing[1,12], Sunil K. Joshi[10,11], Karen A. Ketchum[16], Justin Kirby[33], Iga Kolodziejczak[40], Yelena V. Krutikova[42], Chandan Kumar-Sinha[1,12], Jennifer E. Kyle[5], Priscila M. Lalli[5], Toan Le[19], Jonathan T. Lei[3,4], Ginny Xiaohe Li[1], Qing Kay Li[13], T. Mamie Lih[13], Tao Liu[5], Yin Lu[19], Avi Ma'ayan[37], Micheal J. MacCoss[43], Kiran K. Mangalaparthi[38], D.R. Mani[35], Rahul Mannan[1,12], Monica Mays[20], Peter McGarvey[16], Mehdi Mesri[16], Matthew E. Monroe[5], Ronald J. Moore[5], Alexey I. Nesvizhiskii[1,2], Chelsea Newton[17], James Noyama[36], Kristen Nyce[19], Gilbert S. Omenn[2], Akhilesh Pandey[38], Abhijit Parolia[1,12], Amanda G. Paulovich[44], Vanessa L. Paurus[5], Francesca Petralia[37], Vladislav A. Petyuk[5], Alex Pico[45], Paul D. Piehowski[6], Alexander Pilozzi[19], Pinar O. Eser[35], James C. Pino[5], Daniel A. Polasky[1], Camilo Posso[5], Olga Potapova[39], Marina Prilutskaya[42], Gustavo Rivero[46], Ana I. Robles[16], Karin D. Rodland[5,29], Dan Rohrer[17], Paul Rudnick[47], Tyler J. Sagendorf[5], Shankha Satpathy[35], Athena A. Schepmoes[5], Zhiao Shi[3,4], Yvonne Shutack[33], Richard D. Smith[5], Sandra S. Garcia-Buntley[32], Kelly G. Stratton[5], Bahar Tercan[7], Ratna R. Thangudu[16], Mathangi Thiagarajan[32], Cristina E. Tognon[21], Elie Traer[21,23], Chia-Feng Tsai[5], Jeffrey W. Tyner[26], Negin Vatanian[33], Sudha Venkatachari[32], Miodrag Vucic[34], Chenwei Wang[3,4], Pei Wang[37], Yuefan Wang[13], Karl K. Weitz[5], Bart O. Williams[17], Maciej Wiznerowicz[40], Yuanwei Xu[13], Fengchao Yu[1], Kakhaber Zaalishvili[48], Xu Zhang[16], Hui Zhang[13], Bing Zhang[3,4], Rui Zhao[6], Jasmin Barava[16], Tara Hiltke[16], Henry Rodriguez[16]

1. Department of Pathology, University of Michigan, Ann Arbor, MI 48109, USA.
2. Gilbert S. Omenn Department of Computational Medicine & Bioinformatics, University of Michigan, Ann Arbor, MI 48109, USA
3. Lester and Sue Smith Breast Center, Baylor College of Medicine, Houston, TX 77030, USA
4. Department of Molecular and Human Genetics, Baylor College of Medicine, Houston, TX 77030, USA
5. Biological Sciences Division, Pacific Northwest National Laboratory, Richland, WA 99354, USA
6. Environmental and Molecular Sciences Division, Pacific Northwest National Laboratory, Richland, WA 99354, USA
7. Institute for Systems Biology, Seattle, WA 98109, USA
8. Texas Children's Hospital, Department of Pediatrics, Houson, TX 77030, USA
9. Dan L. Duncan Cancer Center, Baylor College of Medicine, Houston, TX 77030, USA
10. Department of Medicine, Divisions of Hematology & Oncology, Stanford University School of Medicine, Stanford, CA 94305, USA
11. Cancer Institute, Stanford University School of Medicine, Stanford, CA 94305, USA
12. Michigan Center for Translational Pathology, University of Michigan, Ann Arbor, MI 48109, USA
13. Department of Pathology. Johns Hopkins University School of Medicine Baltimore, MD 21287, USA
14. Department of Medicine, Washington University in St Louis, St Louis, MO 63110, USA
15. McDonnell Genome Institute, Washington University in St Louis, St Louis, MO 63108, USA
16. Office of Cancer Clinical Proteomics Research, Division of Cancer Treatment & Diagnosis, National Cancer Institute, Rockville, MD 20850, USA
17. Van Andel Research Institute, Grand Rapids, MI 49503, USA
18. Department of Biochemistry and Molecular & Cellular Biology, Georgetown University Medical Center, Washington, DC 20007, USA
19. ICF, 530 Gaither Road, Rockville, MD 20850, USA

20. University of Kansas Medical Center, The University of Kansas Cancer Center, Kansas City, KS 66160, USA
21. Knight Cancer Institute, Oregon Health & Science University, Portland, OR 97210, USA
22. Department of Pathology, Oregon Health and Science University, Portland, OR 97239, USA
23. Division of Hematology & Medical Oncology, Department of Medicine, Oregon Health & Science University, Portland, OR 97239, USA
24. Division of Oncology, Department of Medicine, Washington University, St. Louis, MO 63108, USA
25. Siteman Cancer Center, Washington University, St. Louis, MO 63108, USA
26. Department of Cell, Developmental & Cancer Biology, Knight Cancer Institute, Oregon Health & Science University, Portland, OR 97239, USA
27. Department of Urology, University of Michigan Medical School, Ann Arbor, MI, USA
28. Howard Hughes Medical Institute, Ann Arbor, MI 48109, USA
29. Department of Cell, Developmental, and Cancer Biology, Oregon Health & Science University, Portland, OR 97221, USA
30. Department of Biomedical Engineering, Oregon Health and Sciences University, Portland, OR 97221, USA
31. School of Public Health, University of Michigan, Ann Arbor, MI 48109, USA.
32. Frederick National Laboratory for Cancer Research, 8560 Progress Dr, Frederick, MD 21701, USA
33. Leidos Biomedical Research Inc., 4912 Research Dr NW, Huntsville, AL 35805, United States
34. Fidelis Research, 44A Sveti Kipriyan Str, Floor 4,Sofia 1799 Bulgaria
35. Broad Institute of MIT and Harvard, 415 Main St, Cambridge, MA 02142
36. University of Pittsburgh Medical Center, 200 Lothrop St. Pittsburgh, PA 15213-2582
37. Icahn School of Medicine at Mount Sinai, 1 Gustave L Levy Pl, New York, NY 10029, United States
38. Department of Laboratory Medicine and Pathology, Mayo Clinic, Rochester, MN 55905, USA.
39. Cureline Human Biospecimen CRO, 6000 Shoreline Ct #102, South San Francisco, CA 94080

40. International Institute for Molecular Oncology, J Krauthofera 23, 60-203 Poznań, Poland
41. University of California, 1001 Health Sciences Road, Irvine CA 92697-3950
42. ProteoGenex, Inc., 6133 Bristol Parkway #157 Culver City, CA 90230
43. University of Washington, 1410 NE Campus Parkway, Seattle, WA 98195
44. Fred Hutch Cancer Center, 1100 Fairview Ave N, Seattle, WA 98109
45. Gladstone Institutes, 1650 Owens St, San Francisco, CA 94158, United States
46. TGH Cancer Center 3 Tampa General Circle Tampa, FL 33606
47. Spectragen Informatics, 4491 Ne North Tolo Rd, Bainbridge Is, WA 98110-3496
48. BioPartners, 3075 Townsgate Rd, Ste 140, Westlake Village, CA 91361, United States
