# Supplementary material for: Integrated proteogenomic and metabolomic profiling of acute myeloid leukemias to identify molecular subtypes and associated therapy targets
Source: Nat Cancer. 2026 Jun 12;7(6):993–1015. doi: 10.1038/s43018-026-01175-6 (PMC13309276; doi:10.1038/s43018-026-01175-6)
Supplement: Supplementary file 2 — Reporting Summary [file 43018_2026_1175_MOESM2_ESM.pdf]

nature portfolio

Corresponding author(s): Marcin Cieslik
Tao Liu
Bing Zhang
Alexey Nesvizhskii

Last updated by author(s): Jan 8, 2026

Reporting Summary

Nature Portfolio wishes to improve the reproducibility of the work that we publish. This form provides structure for consistency and transparency in reporting. For further information on Nature Portfolio policies, see our Editorial Policies and the Editorial Policy Checklist.

Statistics

For all statistical analyses, confirm that the following items are present in the figure legend, table legend, main text, or Methods section.

n/a	Confirmed	
☐	☒	The exact sample size (*n*) for each experimental group/condition, given as a discrete number and unit of measurement
☐	☒	A statement on whether measurements were taken from distinct samples or whether the same sample was measured repeatedly
☐	☒	The statistical test(s) used AND whether they are one- or two-sided<br>*Only common tests should be described solely by name; describe more complex techniques in the Methods section.*
☐	☒	A description of all covariates tested
☐	☒	A description of any assumptions or corrections, such as tests of normality and adjustment for multiple comparisons
☐	☒	A full description of the statistical parameters including central tendency (e.g. means) or other basic estimates (e.g. regression coefficient) AND variation (e.g. standard deviation) or associated estimates of uncertainty (e.g. confidence intervals)
☐	☒	For null hypothesis testing, the test statistic (e.g. *F*, *t*, *r*) with confidence intervals, effect sizes, degrees of freedom and *P* value noted<br>*Give P values as exact values whenever suitable.*
☒	☐	For Bayesian analysis, information on the choice of priors and Markov chain Monte Carlo settings
☒	☐	For hierarchical and complex designs, identification of the appropriate level for tests and full reporting of outcomes
☐	☒	Estimates of effect sizes (e.g. Cohen's *d*, Pearson's *r*), indicating how they were calculated

Our web collection on statistics for biologists contains articles on many of the points above.

Software and code

Policy information about availability of computer code

Data collection	Data collection (metabolomics, lipidomics, proteomics, genomics) was performed as described in the methods. No custom software or methods were used other than the vendor-provided drivers and toolkits to operate the instruments (sequencers, mass-spectrometry, array readers).

Data analysis	CNVEX (https://github.com/mctp/cnvex) R (v3.2.3) (R Development Core Team; https://www.R-project.org) Python (Python Software Foundation; https://www.python.org/) FragPipe (Yu et al.; https://fragpipe.nesvilab.org/) Philosopher (da Veiga Leprevost et al.; https://philosopher.nesvilab.org/) MSFragger (Kong et al.; https://msfragger.nesvilab.org/) PTM-Shepherd (Geiszler et al.; https://ptmshepherd.nesvilab.org/) TMT-Integrator (Djomehri et al.; https://github.com/Nesvilab/TMT-Integrator) ARD-NMF (Tan et al.; https://github.com/getzlab/getzlab-SignatureAnalyzer) CancerSubtypes (Xu et al.; https://www.bioconductor.org/packages/release/bioc/html/CancerSubtypes.html) Limma (Ritchie et al.; https://bioconductor.org/packages/release/bioc/html/limma.html) PTM-SEA (Krug et al.; https://github.com/broadinstitute/ssGSEA2.0) ClusterProfiler (Yu et al.; https://www.bioconductor.org/packages/release/bioc/html/clusterProfiler.html) SCENIC (1.3.1) (Van de Sande et al.; https://scenic.aertslab.org/) Survival (Therne et al.; https://cran.r-project.org/web/packages/survival/index.html) fastICA (1.2-4) (Marchini et al.; https://cran.r-project.org/web/packages/fastICA/index.html) XGBoost (Chen et al.; https://xgboost.readthedocs.io/en/stable/) Graphpad Prism 8 (https://www.graphpad.com/features) PyMOL (2.5.0) (https://www.pymol.org/) MS-DIAL v4.9 (Tsugawa et al. 2020; https://systemsomicslab.github.io/compms/msdial/main.html) Compound Discoverer3.3 (ThermoFisher Scientific; https://mycompounddiscoverer.com/) pmartR (Degnan et al. (2023), Stratton et al. (2019); https://cran.r-project.org/web/packages/pmartR/index.html) FunMap (Shi et al.; https://github.com/bzhanglab/funmap) ICE (Shi et al.; http://ice.zhang-lab.org/) SNFtool (https://github.com/cran/SNFtool). Analysis scripts and notebooks used for this study are open-source and available at https://github.com/Nesvilab/CPTAC_AML.

For manuscripts utilizing custom algorithms or software that are central to the research but not yet described in published literature, software must be made available to editors and reviewers. We strongly encourage code deposition in a community repository (e.g. GitHub). See the Nature Portfolio guidelines for submitting code & software for further information.

Data

Policy information about availability of data

All manuscripts must include a data availability statement. This statement should provide the following information, where applicable:

- Accession codes, unique identifiers, or web links for publicly available datasets
- A description of any restrictions on data availability
- For clinical datasets or third party data, please ensure that the statement adheres to our policy

Harmonized genomic, transcriptomic, proteomics (raw MS files and processed data files of global proteomics and PTMs), metabolomics, lipidomics, methylation, and clinical data files generated for this AML cohort can be accessed via Genomic Data Commons (GDC) at: https://portal.gdc.cancer.gov (Project ID: CPTAC-3). The raw proteomic data and the processed and harmonized proteogenomic data for the study cohort are available through the Proteomic Data Commons (PDC) at https://pdc.cancer.gov under accession numbers accessions and links: PDC000554 https://proteomic.datacommons.cancer.gov/pdc/study/PDC000554, PDC000555 https://proteomic.datacommons.cancer.gov/pdc/study/PDC000555, PDC000556 https://proteomic.datacommons.cancer.gov/pdc/study/PDC000556, PDC000557 https://proteomic.datacommons.cancer.gov/pdc/study/PDC000557, PDC000558 https://proteomic.datacommons.cancer.gov/pdc/study/PDC000558, PDC000559 https://proteomic.datacommons.cancer.gov/pdc/study/PDC000559, PDC000560 https://proteomic.datacommons.cancer.gov/pdc/study/PDC000560, PDC000561 https://proteomic.datacommons.cancer.gov/pdc/study/PDC000561, PDC000562 https://proteomic.datacommons.cancer.gov/pdc/study/PDC000562. The dataset generated and analyzed during this study is available through the Database of Genotypes and Phenotypes (dbGaP) under accession number phs001287.v22.p7, with controlled access to protect participant privacy and in accordance with the informed consent under which the data were collected. Access requests can be submitted through the dbGaP Authorized Access portal (https://dbgap.ncbi.nlm.nih.gov), where they are reviewed by the NCI DAC (NCIDAC@mail.nih.gov), with approval typically granted within 4–6 weeks to qualified researchers who agree to the Data Use Certification terms. Histopathology images can be accessed via The Cancer Imaging Archive (TCIA) at https://doi.org/10.7937/TCIA.2019.B6FOE619, and the Imaging Data Commons at https://portal.imaging.datacommons.cancer.gov/explore/filters/?collection_id=CPTAC&collection_id=cptac_aml. Source Data Files have been provided for all figures and extended data figures. All other processed data are provided as part of Supplementary Tables.

Research involving human participants, their data, or biological material

Policy information about studies with human participants or human data. See also policy information about sex, gender (identity/presentation), and sexual orientation and race, ethnicity and racism.

Reporting on sex and gender	All manuscript's analyses are based on the genetic sex inferred from sequencing data, cross-references with the reported sex in the clinical records. Due to sample size no sex-specific results are reported.
Reporting on race, ethnicity, or other socially relevant groupings	The manuscript does not report on socially relevant groupings of individuals other than the patients country of origin which is mostly used as a technical (batch) covariate.
Population characteristics	All samples are from patients with acute myeloid leukemia (or very closely related diseases) which were not previously treated or diagnosed with another malignancy.
Recruitment	Samples from multiple tissue collection sites collected from consented patients diagnosed with AML must meet a minimum of 5.0 x 106 cells to a maximum of 1.0 x 107 cells per vial. Blasts comprise >20% of neoplastic cells in the submitted bone marrow aspirate (preferred) and/or >50% of neoplastic cells in the submitted peripheral blood.
Ethics oversight	National Cancer Institute (NCI) / Clinical Proteomic Tumor Analysis Consortium (CPTAC)

Note that full information on the approval of the study protocol must also be provided in the manuscript.

Field-specific reporting

Please select the one below that is the best fit for your research. If you are not sure, read the appropriate sections before making your selection.

☒ Life sciences ☐ Behavioural & social sciences ☐ Ecological, evolutionary & environmental sciences

For a reference copy of the document with all sections, see nature.com/documents/nr-reporting-summary-flat.pdf

Life sciences study design

All studies must disclose on these points even when the disclosure is negative.

Sample size	The sample size was not statistically pre-determined.
Data exclusions	No samples meeting technical (pre-analytical or analytical) quality controls were excluded from analyses.
Replication	All experiments were performed in triplicates or more. Technical reproducibility was assessed through parallel technical runs of a subset of samples. Concordance of bone-marrow and peripheral blood derived AML was performed. Results were confirmed whenever appropriate in other AML cohort and datasets such a Beat-AML.
Randomization	No randomization was performed
Blinding	No blinding was performed

Reporting for specific materials, systems and methods

We require information from authors about some types of materials, experimental systems and methods used in many studies. Here, indicate whether each material, system or method listed is relevant to your study. If you are not sure if a list item applies to your research, read the appropriate section before selecting a response.

Materials & experimental systems

n/a	Involved in the study	
☐	☒	Antibodies
☐	☒	Eukaryotic cell lines
☒	☐	Palaeontology and archaeology
☒	☐	Animals and other organisms
☐	☒	Clinical data
☒	☐	Dual use research of concern
☒	☐	Plants

Methods

n/a	Involved in the study	
☒	☐	ChIP-seq
☒	☐	Flow cytometry
☒	☐	MRI-based neuroimaging

Antibodies

Antibodies used	anti-MTA1 (D17G10) antibody<br>Cell Signaling Technologies<br>Catalog: 5646<br>immunohistochemistry: 1:200-1:500
Validation	Rabbit IgG, all CST antibodies are validated per manufacturer description and guarantee https://www.cellsignal.com/about-us/our-approach-process/cst-antibody-performance-guarantee https://www.cellsignal.com/products/primary-antibodies/mta1-d17g10-rabbit-mab/5646

Eukaryotic cell lines

Policy information about cell lines and Sex and Gender in Research

Cell line source(s)	The cell lines used in this study include CAKI-1 (ATCC, Catalog: HTB-46), NB-1 (Sekisui XenoTech, LLC, Catalog: JCRB0621 NB-1), HDMYZ (DSMZ, Catalog: ACC 346), SIGM5 (DSMZ, Catalog: ACC 468), MONO-MAC-6 (DSMZ, Catalog: ACC 124), SKM-1 (DSMZ, Catalog: ACC 547), HL-60 (ATCC, Catalog: CCL-240), MOLM-14 (courtesy of Yoshinobu Matsuo's Lab), HEK 293T/17 (courtesy of Brian Druker's Lab), and Quizartinib-resistant MOLM-14 cell (courtesy of Elie Traer's Lab).
Authentication	Cell lines were authenticated by the biobanks (ATCC DSMZ) or provided by the collaborating laboratories.

Mycoplasma contamination | Cell lines were not tested for mycoplasma contamination.

Commonly misidentified lines (See ICLAC register) | No commonly misidentified cell lines were used in the study.

Clinical data

Policy information about clinical studies

All manuscripts should comply with the ICMJE guidelines for publication of clinical research and a completed CONSORT checklist must be included with all submissions.

Clinical trial registration	N/A
Study protocol	N/A
Data collection	All clinical data was abstracted retrospectively from the clinical record by the tissue source sites (CPTAC)
Outcomes	This study is not a clinical trial and does not report on primary or secondary clinical outcomes.

Plants

Seed stocks	N/A
Novel plant genotypes	N/A
Authentication	N/A
