# Supplementary material for: Integrated proteogenomic and metabolomic profiling of acute myeloid leukemias to identify molecular subtypes and associated therapy targets
Source: Nat Cancer. 2026 Jun 12;7(6):993–1015. doi: 10.1038/s43018-026-01175-6 (PMC13309276; doi:10.1038/s43018-026-01175-6)
Supplement: Supplementary file 10 — Unprocessed western blots and/or gels. [file 43018_2026_1175_MOESM10_ESM.pdf]

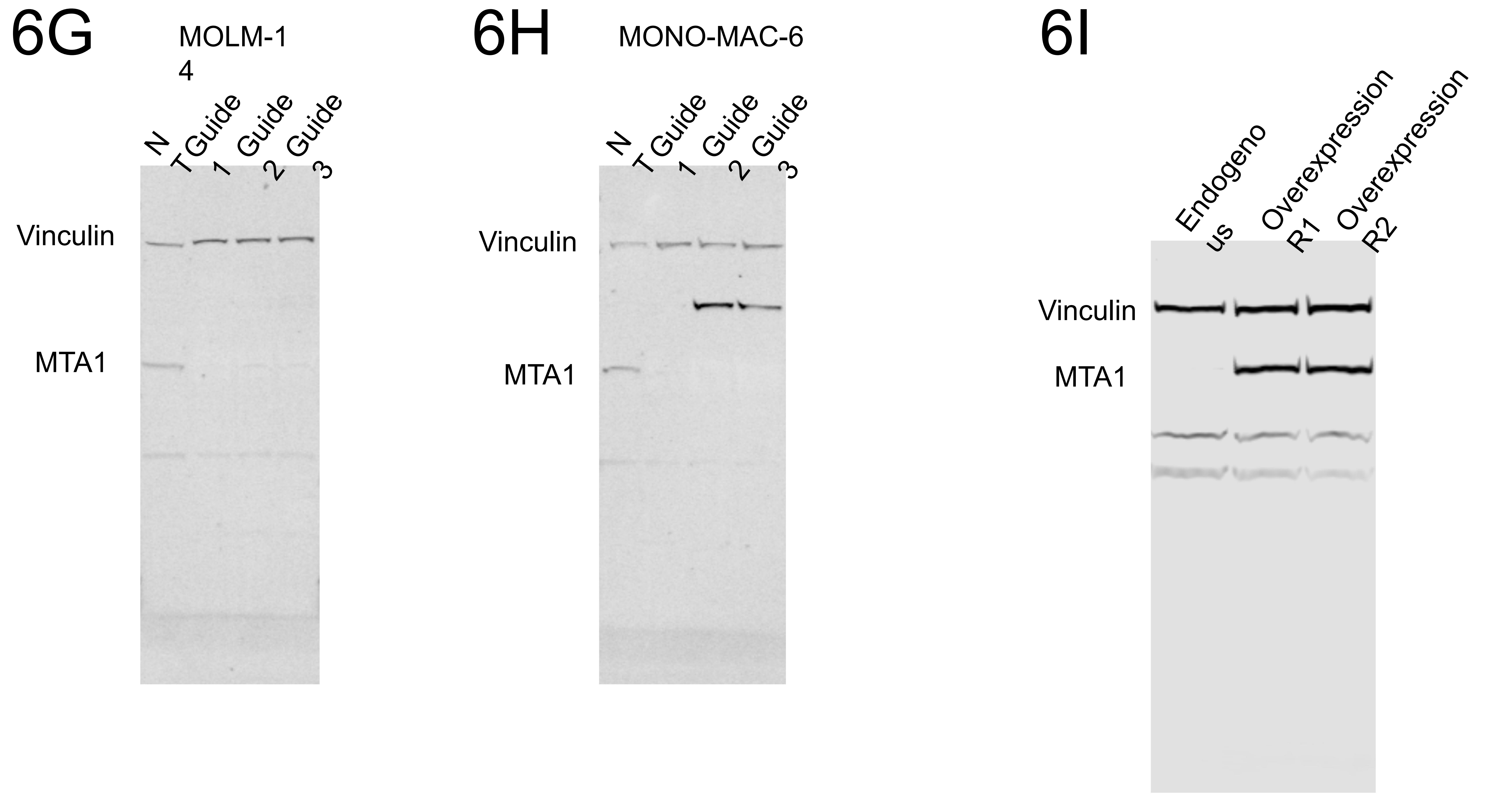
6G
MOLM-1
4
N
T
Guide
1
Guide
2
Guide
3
Vinculin
MTA1
6H
MONO-MAC-6
N
T
Guide
1
Guide
2
Guide
3
Vinculin
MTA1
6I
Endogeno
us
Overexpression
R1
Overexpression
R2
Vinculin
MTA1
